# Permutation Entropy as a Universal Disorder Criterion: How Disorders at Different Scale Levels Are Manifestations of the Same Underlying Principle

**DOI:** 10.3390/e23121701

**Published:** 2021-12-20

**Authors:** Rutger Goekoop, Roy de Kleijn

**Affiliations:** 1 Parnassia Group, PsyQ Parnassia Academy, Department of Anxiety Disorders, Early Detection and Intervention Team (EDIT), Lijnbaan 4, 2512 VA Den Haag, The Netherlands; 2 Cognitive Psychology Unit, Institute of Psychology & Leiden Institute for Brain and Cognition, Leiden University, Wassenaarseweg 52, 2333 AK Leiden, The Netherlands; kleijnrde@fsw.leidenuniv.nl

**Keywords:** permutation entropy, disorder, stress, allostatic (hub) overload, cascading failure, disease, hierarchical control systems, active inference, free energy principle, critical slowing down

## Abstract

What do bacteria, cells, organs, people, and social communities have in common? At first sight, perhaps not much. They involve totally different agents and scale levels of observation. On second thought, however, perhaps they share everything. A growing body of literature suggests that living systems at different scale levels of observation follow the same architectural principles and process information in similar ways. Moreover, such systems appear to respond in similar ways to rising levels of stress, especially when stress levels approach near-lethal levels. To explain such communalities, we argue that all organisms (including humans) can be modeled as hierarchical Bayesian controls systems that are governed by the same biophysical principles. Such systems show generic changes when taxed beyond their ability to correct for environmental disturbances. Without exception, stressed organisms show rising levels of ‘disorder’ (randomness, unpredictability) in internal message passing and overt behavior. We argue that such changes can be explained by a collapse of allostatic (high-level integrative) control, which normally synchronizes activity of the various components of a living system to produce order. The selective overload and cascading failure of highly connected (hub) nodes flattens hierarchical control, producing maladaptive behavior. Thus, we present a theory according to which organic concepts such as stress, a loss of control, disorder, disease, and death can be operationalized in biophysical terms that apply to all scale levels of organization. Given the presumed universality of this mechanism, ‘losing control’ appears to involve the same process anywhere, whether involving bacteria succumbing to an antibiotic agent, people suffering from physical or mental disorders, or social systems slipping into warfare. On a practical note, measures of disorder may serve as early warning signs of system failure even when catastrophic failure is still some distance away.

## 1. A Short History on Stress Tolerance Studies in Different Organisms

**Figure 1:**
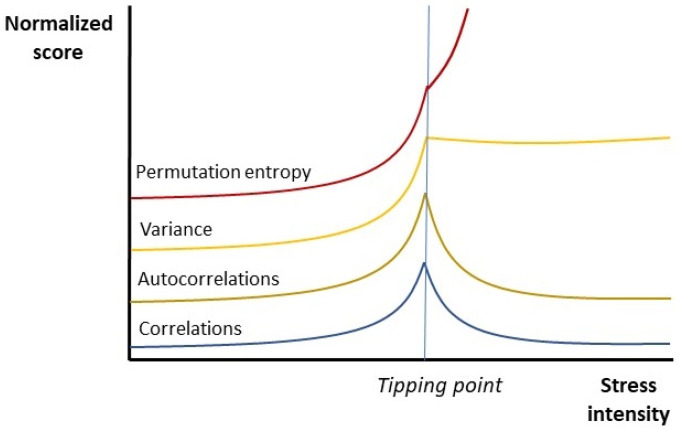
Universal changes in signal transduction of living systems under rising levels of stress. *Just before living systems undergo a sudden phase transition (a tipping point, e.g., disease or death), they show characteristic changes in internal signal transduction that may serve as early warning signs for system failure. As can be observed from this schematic figure, (auto)correlations between system components increase prior to the tipping point and decrease afterward, whereas variance increases and remains high (after [13]). A generic cause of such changes has so far remained unclear. In this paper, we argue that these changes are incorporated by a single variable (permutation entropy, see below), which may provide insights into a universal mechanism that underlies critical transitions in living systems*.

For a long time, it was believed that different organisms respond in different ways to environmental challenges. This assumption is understandable, since stress responses in bacteria, fish, birds, or mammals involve totally different genetic and neural pathways. When ignoring the details of a particular stress response and observing the whole of system dynamics at a slightly more abstract level, however, such differences disappear. No matter what type of organism is studied, its response to unfavorable environmental conditions is essentially the same: the various components that constitute the organism (such as genes, proteins, metabolites, neurons, or brain regions) increasingly synchronize their responses and assume a larger number of different values [1,2]. In other words, the strength of correlations between system components increases, as so does the variance. Meanwhile, system components remain within the same state for longer periods of time, causing the values of these components to correlate more strongly with their previous values (‘autocorrelations’). This happens up to a discrete point, after which synchronization decreases but variance remains high. Such ‘tipping points’ usually correspond to the onset of disease or the death of the organism (Figure 1). This peculiar phenomenon has been rediscovered many times since the 1980s. Examples include an impressive range of organisms and types of stressors, from bacteria succumbing to antibiotic stressors and plants fighting conditions of severe drought to insects, reptiles, birds, and mammals that struggle under all sorts of unfavorable conditions [1]. In humans, the same dynamics can be observed in cardiac muscle cells prior to myocardial infarction, asthmatic attacks in patients with obstructive pulmonary disease, and neuronal activity prior to cardiac arrhythmias and epileptic seizures [2]. In addition to physical disorders, similar changes have been observed in self-reported mental states of patients with different forms of acute mental illness, such as major depression, bipolar disorders, or psychosis [3,4,5]. This generic response to environmental challenges seems to be independent of the spatial scale level of observation. It has been observed to govern the dynamics of molecules, genes, different cell types, tissues, organs and whole organisms, food webs, stock markets, and entire ecosystems [2]. Typically, just before the tipping point occurs, the system becomes slow to recover from environmental perturbations, which is why this phenomenon is sometimes referred to as ‘critical slowing down’ (CSD) [6,7]. CSD has been confirmed in different fields of science, although knowledge of this phenomenon still seems to be largely restricted to the physical rather than biological sciences [8,9]. There may be several factors that contribute to CSD, but a generic mechanism that underlies CSD at multiple scale levels so far remains elusive. Critical slowing down may be due to a gradual increase in the number and strength of recurrent connections between system components (e.g., computers, genes, neurons, or people) [10]. Such components continuously enforce each other’s activity, for which reason it will take longer for the system to quiet down after initial perturbation (‘hysteresis’ or slowing down: this would explain the increase in autocorrelations). A gradual increase in the number and strength of local connections decreases the number of network clusters (communities of connected nodes) until, at some discrete point, only a few additional connections are required to link all network clusters together into one giant connected component [11]. At that point, only a small increase in local connectivity is sufficient to produce an abrupt change in global network activity: a phase transition [12]. Despite such valuable insights, however, it has so far remained unclear what causes the connectivity and variance of system components to increase prior to a tipping point or to decrease after the tipping point has been reached.

Rising levels of stress do not only cause universal changes in internal signal transduction of living systems. The content of their behavior also changes in an apparently universal way. When stress levels approach near-lethal levels, organisms shift their behavior from so called ‘slow’ to ‘fast’ behavioral policies [14]. This means they are less prone to spending time and energy on caring for each other and future generations (e.g., reproduction and parental investment). Instead, they become more focused on energy economy and self-preservation (e.g., aggression and maternal cannibalism). Behavior also shifts from long-term strategies (e.g., storing food, stacking fat) toward more short-term strategies (e.g., eating food, burning fat). Physiologically, such changes coincide with a shift back from more sophisticated, ‘goal-directed’ forms of behavior (such as navigating mazes in order to locate a food source) to relatively simple, habitual forms behavior (such as feeding, fighting, or fleeing) [15,16]. In other words, the organism’s behavior becomes more focused on managing basic challenges that are currently at hand, rather than considering complex and possibly long-term scenarios. Such changes have previously been explained by a need of organisms to redistribute scarce amounts of energy and resources to their most primary processes [17,18]. In this view, organisms can be modeled as regulatory systems with a hierarchical structure, in which higher and lower systems work together to produce stability [19]. When a lower-level system fails to stabilize the organism, a higher-level system will take over to nonetheless secure stability. The lower regulatory levels are called ‘homeostatic’ systems, since they are concerned with the relatively simple task of maintaining some state of the system within some narrowly defined limits (e.g., raising insulin or glucagon levels to keep glucose levels within certain limits). Higher-level systems are called ‘allostatic’ systems, since they are concerned with maintaining “stability through change” [19]. This usually involves more elaborate forms of behavior that will secure stability via a detour (e.g., navigating a complex environment to locate a food source, the ingestion of which will eventually raise glucose levels) [20]. To explain the observed changes in behavioral policies of organisms under stress, it has been proposed that stress induces an ‘allostatic overload’, i.e., a failure of higher-level (allostatic) systems that require a lot of energy to secure stability, leaving the more energy-efficient lower-level (homeostatic) systems to fend for themselves. Although this sounds intuitively appealing, the mechanism behind allostatic overload, as well as the way in which this mechanism relates to the observed changes in behavioral policies, has so far remained unclear. In this paper, we offer an explanation of these changes that has its footings in first principles in biophysics and control theory. Below, we first discuss the common stress response in somewhat more detail. After that, we discuss a consensus view on the structure and function of living systems that results from the integration of network theory, systems biology, and the free energy principle [21]. Departing from this framework, we then propose a generic mechanism that explains the characteristic changes in signal transduction and overt behavior of living systems under high levels of stress.

## 2. Disorder as a Common Response of Organisms to High Levels of Stress

**Figure 2:**
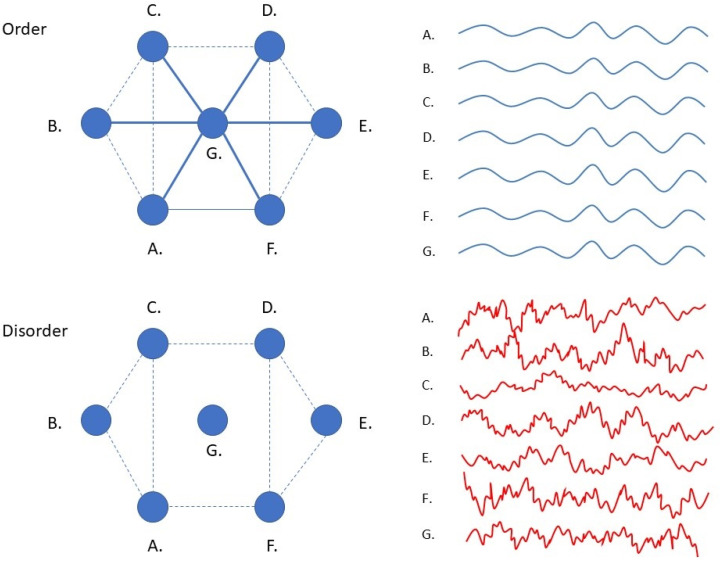
Increased disorder (permutation entropy) may be due to a loss of regulatory connections. *The emergence of disorder in timeseries may be due to the loss of regulatory connections that normally synchronize system components (e.g., genes, neurons) to produce order. In this figure, G is a regulatory (hub) node with many connections that synchronize the timeseries of node A–F. The loss of regulatory connections may cause nodes A–F to show autonomous (unsynchronized and, hence, disordered) behavior. The reason for this loss of regulatory connections has so far remained unclear*.

In a seminal study, Zhu et al. showed that bacteria of different species respond in a similar fashion to antibiotic stressors [22]. Although bacterial stress responses include many different genetic pathways that depend on the type of stressor and the bacterial species involved, a generic stress response could nonetheless be observed when considering the whole system dynamics (i.e., when observing the whole gene transcription activity as measured in terms of differential mRNA expression in time). When antibiotic concentrations approach near-lethal levels, this causes a decrease in the number of statistical dependencies that normally exist between the genes of bacteria (correlations decrease, but variance remains high). This loss of coherence in gene expression was observed to increase the amount of randomness of the timeseries that describe differential gene expression in time. Such ‘disorder’ can be expressed in terms of a statistical quantity called permutation entropy, which is a measure of the amount of randomness that can be observed in the covariance patterns the describe the relationships between the various components of a system (Box 1). Zhu et al. noted that the observed rise in disorder scores resulted from large-amplitude changes that were produced by independently responding genes, and that this independence may result from of a loss of regulatory connections that normally synchronize gene activity to produce order (Figure 2) [22]. As it turns out, permutation entropy levels in the timeseries of bacterial gene expression predict bacterial fitness (defined as the growth and survival rates of bacteria). Such predictions can be made with superior accuracy when compared to standard techniques that rely on the expression profiles of specific genetic pathways. This allows doctors to select antibiotics that are effective in treating certain types of bacterial infections, even when the specific genetic pathways involved in a particular bacterial stress response are not fully known.

Box 1 **Permutation entropy explained.**

Permutation entropy is a measure of the amount of disorder, unpredictability, randomness, or information content of a timeseries [23]. In calculating this measure, the values of successive timepoints are examined for predictable patterns by ordering them in partitions of prespecified length n (e.g., in case *n* = 3, the timeseries (1 9 3 5 2 7) will yield the partitions [1 9 3], [9 3 5], [3 5 2], etc.). The values of each partition are then placed in ascending order (e.g., for [1 9 3], the ascending order is [1 3 9]), and each value of the ordered partition is then assigned the logical code [0 1 2], depending on its position in the ascending sequence (e.g., 1 = 0, 3 = 1, 9 = 2). The full timeseries is then recoded according to this code table (e.g., the partition [1 9 3] is recoded into [0 2 1], [9 3 5] is recoded into [2 0 1], [3 5 2] is recoded into [1 2 0], etc.). Such logical reorderings of numbers are called permutations. The relative frequency *p* (π) of all *n*! permutations π of order *n* is then calculated, which expresses the probability of occurrence of some permutation with respect to all others in the timeseries. The permutation entropy is then calculated, which is a measure of the amount of patternlessness or randomness in the timeseries. This is done as follows:

H(n)=−∑p(π)ln p(π),

where the sum is run across all *n*! permutations π of order *n*. From this formula, it can be seen that *H*(*n*) lies in between 0 and 1, with the value 0 indicating a completely logically ordered timeseries of either ascending or descending values and the value 1 meaning complete randomness.

The calculation of permutation entropy scores requires only few parameters and can be done quickly. A single score can be calculated for a single timeseries or set of timeseries at once, enabling a study of global signal intensity changes within organisms (e.g., differential mRNA expression in time, or activity changes in brain regions), as well as their overt behavior as a function of stress levels [22]. To study a set of timeseries at once, PE can be expressed as the natural logarithm of a glasso-regularized empirical correlation matrix M, which contains the partial correlation coefficients of all statistical relationship between the components of a system [22]. PE is then expressed as follows:

$$H=ln\left|M_{\rho }\right|,$$

where || denotes the regularization, and ρ signifies the regularization strength. Crucially, permutation entropy can be calculated not only for timeseries, but also for a single timepoint (stp), in which case the cross-sectional (snapshot) level of disorder of the system can be expressed as a single value [22,24].

$$H_{stp}=ln\left(\sigma^{2}\right),$$

where *σ*^2^ denotes the variance of the distribution across all measured variables.

The traditional PE measure as explained above does not take the amplitude (or weight) of signal changes into account. Additionally, it is insensitive to signal changes at different temporal scale levels (i.e., high- versus low-frequency components) and highly sensitive to differences in the length of a timeseries and noise artefacts. For this reason, several refinements have been proposed of the original PE measure, which involve calculating weighted PE scores that are compared to white noise (pure randomness) across multiple (coarse grained) temporal scale levels. This refined multiscale reverse weighted (RMSRW) permutation entropy measure can handle noisy timeseries of different lengths, as well as signal changes at different scale levels [25]. By incorporating amplitude, variance, and temporal autocorrelations into a single value, RMSRW-PE covers all aspects that are considered typical hallmarks of critical slowing down (CSD). This means that living systems become increasingly ‘disordered’ prior to their failure, which we argue results from a loss of integrative regulatory connections that normally synchronize system components to produce order (see text). Throughout the rest of this paper, we use the terms PE and ‘disorder’ interchangeably as a more parsimonious term to refer to signal changes in stressed systems prior to their collapse.

Since (weighted) permutation entropy is a measure of global system dynamics, it incorporates the previously observed changes in correlation strength, variance, and (auto)correlations that are considered typical hallmarks of critical slowing down (Box 1). The permutation entropy measure appears to have comparable usability to the traditional measures of CSD. For instance, rising levels of permutation entropy are observed in living systems across all scale levels of biological organization, from genes and individual cells to tissues, organs, organisms, and social communities [26]: the death of a single bacterium follows the same dynamics as the collapse of a multicellular organism, populations of organisms, or entire ecosystems [27]. The increase in disorder levels affects both internal signal transduction and the outwardly observable behavior of organisms. For instance, fruit flies show erratic flying patterns when air pollution levels are high [28]. Water fleas, mussels, fish, dolphins, and whales show increasingly disordered swimming patterns when water quality deteriorates [29,30,31]. Human locomotion patterns show signs of increased randomness when stressed [32]. Like traditional measures of CSD, permutation entropy is able to predict the onset of tipping points in living systems, which signal the sudden onset of disease (or death). For example, bacteria succumbing to an antibiotic stressor, plants dying from a lengthy drought, or the bleaching of coral in deteriorating environments are typically discrete events that can be predicted by elevated levels of PE. Such findings have inspired ideas to use permutation entropy as part of an early warning system to monitor plant and animal welfare [29,30,31,33]. In humans, early warning signs of system failure typically precede the (sudden) onset of physical or mental illness [3,4,5,26]. Such knowledge is gradually making its way to medical practice. Permutation entropy levels can predict the onset of blood infections [34] and the spread of infectious disease throughout human populations [35]. In cardiology, neurology, and psychiatry, early warning signs for epileptic seizures, cardiac arrhythmias, and major depressive or psychotic disorders may allow for timely countermeasures [5,26]. Such observations underscore the practical value of ‘disorder’ as an early warning sign and warrants a further look into optimal descriptors of this phenomenon, as well as its possible causes.

The idea that permutation entropy can be used as a single parsimonious measure of signal changes in struggling systems has practical consequences in the sense that it reduces the complexity of calculations. More importantly, however, this conceptual step may help to gain a better understanding of the possible mechanisms that underlie CSD. On the one hand, the presence of generic early warning signs in struggling systems may just be a coincidence, with many different causes of disorder loading onto a single quantity (permutation entropy) that is so generic that it fails to say anything useful about living systems. On the other hand, such similarities may suggest a common biophysical principle that underlies disorder at different scale levels of organization [27,36]. Below, we argue for the latter position by showing that similar biophysical rules govern the structure and function of living systems at different scale levels of organization. We show that living systems are hierarchically organized network structures in which highly connective components (hubs) maintain high-level allostatic control. We then show that stress can be equated to variational free energy under the free-energy principle [37,38] and that high levels of stress (free energy) specifically cause the most connective nodes in a network (hubs) to overload and fail, since these are the first to reach their limits of free-energy dissipation. Since hubs keep the various components of a system together and synchronized (like horse cart drivers keeping a team of horses in check), the failure of such structures produces desynchronization and disorder, including the generic early warning signs as described above. We argue that a loss of (allostatic) control by key connective structures is not necessarily restricted to living systems, but may reflect a universal feature of open dissipative systems that are loaded up with free energy beyond their capacity to dissipate it back to the environment. We conclude by showing how the proposed disorder concept may apply to disease processes in general and to the human situation in particular.

## 3. Organisms as Control Systems

**Figure 3:**
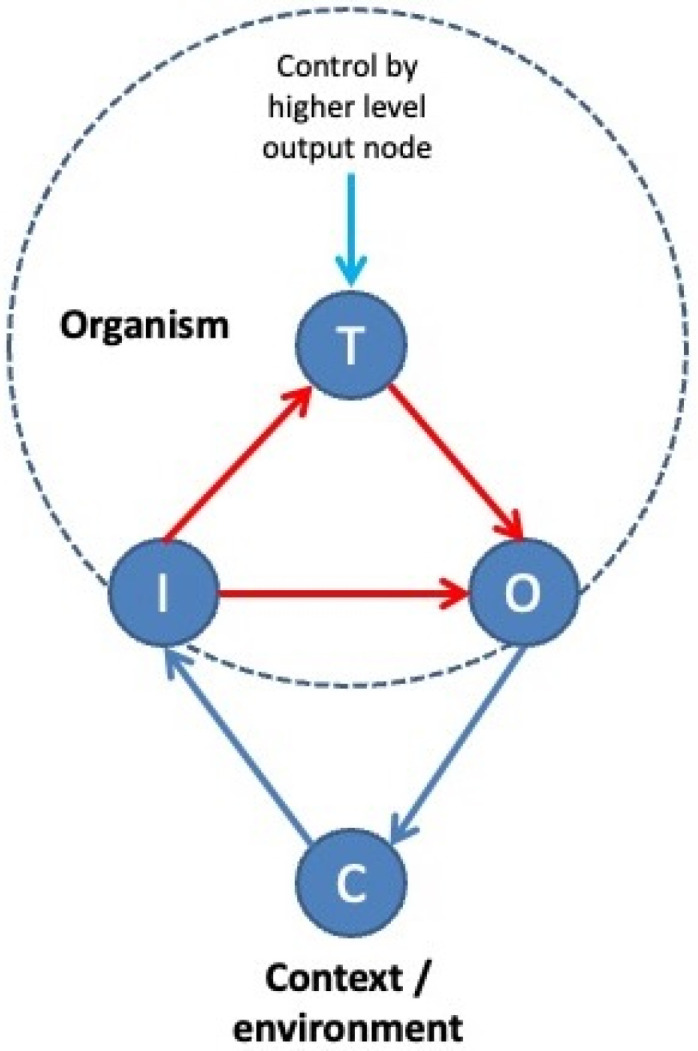
Organisms as control systems. *In a very simple model, organisms can be seen as controls systems with an input (I), throughput (T, reference value), and output compartment that interacts with the environment (context, C). The difference between the input (e.g., temperature) and reference value (e.g., the thermostat) is called the ‘error’, which is transferred onto the output module to generate an action that changes the environment. This in turn changes the input to the organism, and the cycle repeats. Thus, organisms iteratively seek out (or create) environments that fit their reference values. Complex (sets of) reference values are called ‘world models’. These encode a complex set of environmental circumstances representing an optimal econiche (see text)*.

Woodlice keep on running around erratically until the air that surrounds them approaches a humidity level near 100%. Only then do they truly come to rest, which is why we find these creatures in all sorts of nooks and crannies. Woodlice do not know exactly where to find a nice and wet place in which they can safely retreat from the dangers of desiccation: they just keep on running around until they stumble across a suitable spot, after which the ‘running faucet’ is screwed shut. This mechanism has much in common with the way in which a central heating system works. Such systems have thermostats that indicate the desired temperature (e.g., 22 °C, a ‘setpoint’), sensors that indicate the actual temperature (e.g., 18 °C) and heating elements that produce heat. The difference (4 °C) between the desired and the actual temperature is sent to the heater that heats up the environment until room temperature reaches the preset value. At that very moment, the difference (‘error’) is zero, and the heater shuts down. All organisms, including humans, turn out to follow this same principle: we are ‘control systems’ that try to minimize the difference between our ‘setpoints’ and the actual state of the environment [39]. It is just that our setpoints are more elaborate and describe several more desirable states than just ambient temperature (e.g., partners, jobs, and social positions). Together, the total collection of our setpoints describes our preferential ‘econiches’: spots on the planet and in our society where we like to be and where we will eventually end up provided these niches are encoded correctly and the right actions are performed in order to reach these places (Figure 3).

## 4. Active Inference

In reality, things are a bit more complicated: our thermostats do not merely indicate which states we like to experience. They indicate which states we *expect to occur* at some point in the future. That means they encode predictions, or predictive models of our environment. This still resembles a thermostat in some way, since one may wonder whether such devices actually indicate what temperature we like, or whether the preset value of 22 °C actually represents a prediction of what room temperature will be, provided the system will keep on running indefinitely. In fact, all setpoints can be construed as predictions, and many setpoints together as predictive models of our inner and outer worlds or preferential niches. Such multifaceted models are called ‘world models’ [40]. The difference between the world that we perceive and our predictive models of that world is called a ‘prediction error’ [41,42]. This is a measure that indicates how ‘surprising’ a certain observation or outcome is, given that outcomes may deviate from predictions. For instance, a frog that is suddenly thrown into a pool of hot water will show a lot of prediction error. Such error provides an estimate of the degree to which its predictive models deviate from the way it perceives the world. In living systems, prediction errors trigger actions that are aimed at reducing the prediction error itself (e.g., the frog will start a struggle to escape its unpleasant surroundings and return to safer grounds). This happens because such actions change the external world (e.g., ambient temperature drops when the frog moves out of the pool), which in turn changes the organism’s perception of that world, which then reduces or increases prediction errors that induce actions, after which the cycle repeats (Figure 3). Action is, therefore, a way to vary prediction errors and test the ‘fitness’ of a world model.

It turns out that prediction errors are not only used to induce action (as in central heating systems), but also to adjust the models (thermostat settings) themselves: a process called ‘belief updating’. This involves a process where the ‘weights’ of the connections between the various elements that constitute the predictive system are altered as a function of prediction error [43]. Thus, belief updating is a form of learning or adaptation, which allows organisms to meet environmental conditions halfway. For instance, the same frog will show less prediction error and remain exactly where it is when put in a pool of cool water that is gradually warmed to unpleasant levels, since it now has the time to adjust its predictive models. The iterative loop of trial (action) and learning from prediction error (belief updating) is called ‘active inference’ [42,44]. This is a process by which organisms are actively foraging their environments for novel experiences that may be counterfactual to (or falsify) their conjectures of the world, after which the most unrefuted model is selected as the most plausible explanation of the observed events [37]. This is sometimes compared to organisms as little scientists [45], although active inference more generally refers to a circular process of inference (niche modeling) and action (niche exploration and active niche construction) [46,47].

**Figure 4:**
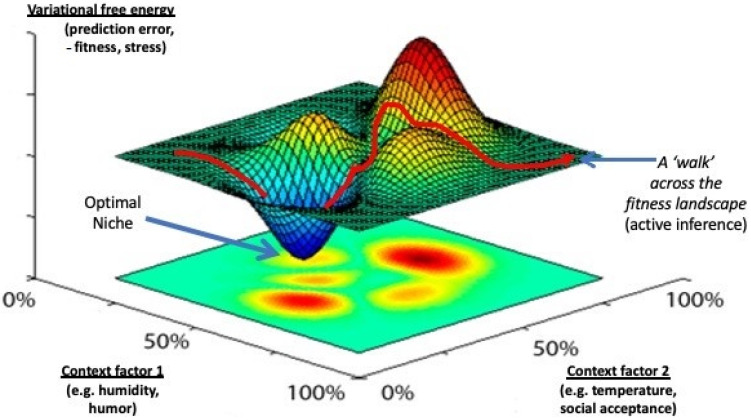
Active inference pictured as organisms exploring a free-energy (model fitness) landscape. *In biology, organisms are said to be involved in niche exploration and active niche construction to occupy econiches that optimize their chances of survival and reproduction (niche exploitation). Active inference theory can be seen as a way to describe this process in biophysical terms. According to this theory, organisms use action to change their environments (e.g., digging in or building a shelter), which in turn alters their perception of the world (e.g., a rise in humidity levels). This altered perception produces a different fit with the organism’s predictive models of the world (alters prediction error), which can be expressed as a change in the theoretical quantity of (variational) free energy. According to the free-energy principle, action (niche exploration and construction) and belief updating (model adjustment) serve to minimize mean variational free energy (produce high average model fitness), allowing organisms to find a low-energy stable state that corresponds to the concept of ‘homeostasis’ in biology. Approaching or occupying optimal econiches, therefore, ensures thermodynamic stability (survival). In this respect, organisms that seek optimal econiches are like ping-pong balls that actively try to roll into pits that correspond to the lowest possible levels of free energy across time (this is called a ‘gradient descent’ on a free-energy plot [55]). In this figure, the vertical axis represents the free energy levels of some organism (prediction error, negative model fitness). The horizontal axes represent environmental conditions (i.e., econiches), which are limited to only two conditions in this example, since we have difficulty imagining organisms navigating multidimensional state spaces (i.e., complex econiches). The various peaks and valleys together form an energy ‘landscape’ (although ‘seascape’ might be more appropriate, since environmental conditions change continuously). Valleys in this seascape represent areas with relatively low (variational) free-energy levels, which correspond to more optimal environmental niches. Active inference is a process by which organisms are continuously improving their internal map of the sea (inference) by actively exploring its surface (niche exploration) and making some ripples of their own (niche construction) to eventually make for the shallowest waters (econiches) where they can remain intact (survive) and reproduce (exploit their niche). In evolutionary biology, similar diagrams are used in which the vertical axis represents ‘reproductive fitness’, which is often defined in terms of the (relative) number of offspring or copies of some gene. In contrast, local or ‘instantaneous fitness’ (prediction error) may be a more proximal measure of biological fitness than gene frequencies or the number of offspring, since the latter measures are counted post hoc. The two can easily be converted into each other,* e.g., *by defining reproductive fitness as the integral of local model fitness (prediction error, homeostasis) across all econiches encountered by the organism and its offspring across some period of time (e.g., the lifespan)*.

In a seminal paper, Karl Friston used insights from Bayesian information theory to show that prediction error (under some circumstances) is equal to the mean amount of ‘variational free energy’ across time of a living system, such as a cell or a brain [48]. This means that when organisms try to iteratively reduce their prediction errors through active inference, they are actually trying to reduce their free-energy levels across longer timespans. In this respect, they are not much different from crystals in which ions arrange themselves into highly ordered patterns, despite the fact that all objects in this universe need to obey the second law of thermodynamics (which states that they must seek a state of maximum disorder, i.e., high entropy). For quite some time, it was thought that crystals violated the second law of thermodynamics, until it was discovered that crystallization produces heat that dissipates into the environment, producing a global increase in entropy (and free energy) levels [49]. Additionally, the ordering of ions into neatly arranged lattices in many cases allows water molecules to move more freely through the system, which adds to the global amount of disorder (and free energy) of the universe. Thus, scholars realized that objects may arrange themselves *locally* into more ordered (low-entropy) states as long as this allows for a *global* increase in entropy and free energy. Despite the necessity that everything in nature eventually needs to revert to a state of high disorder, living systems have found a way in which they can maintain their circumscribed form and stable state (i.e., order) at least for some period of time, by having found the most efficient way of losing (dissipating) their free energy to the environment, which is to reduce prediction error [37,50,51]. Similarly, an organism can be compared to a ping-pong ball that rolls into a pit in order to keep its potential (free) energy as low as possible: that ball simply has no choice, since it needs to obey the second law of thermodynamics, which states that any object may seek a local state of low free energy and entropy (the bottom of the pit) as long as this leads to a global increase in entropy levels of the universe (in this case, the act of rolling into the pit increases the global freedom of the individual molecules of the ball in the form of heat, which subsequently dissipates into the environment [52]). In living systems, the basin of the pit corresponds to a state of low entropy (prediction error or variational free energy) that is called ‘homeostasis’ [37]. Active inference can, therefore, be seen as a walk across a free-energy landscape, in which organisms actively try to roll into pits of low variational free energy that represent high levels of niche model ‘fitness’ (homeostasis) (Figure 4). In most cases, such low-energy states correspond to organisms occupying their locally optimal econiches. The whole process of seeking stability through change thus follows from the basic laws of thermodynamics [51]. Friston has found a series of equations with which to describe this process that do not only apply to life in general, but to all objects in this universe that are required to dissipate their free-energy levels as efficiently as possible [53]: a true ‘Theory of Every Thing’ [54]. In a way, this theory says something we already knew for quite some time: by actively searching for optimal niches (minimizing prediction error), living systems can reach homeostasis (a stable state of low mean variational free energy) and survive (remain intact). The novelty is that we now have mathematical equations with which to describe this process, which may apply to any object in this universe.

## 5. Organisms as Hierarchical Bayesian Control Systems

**Figure 5:**
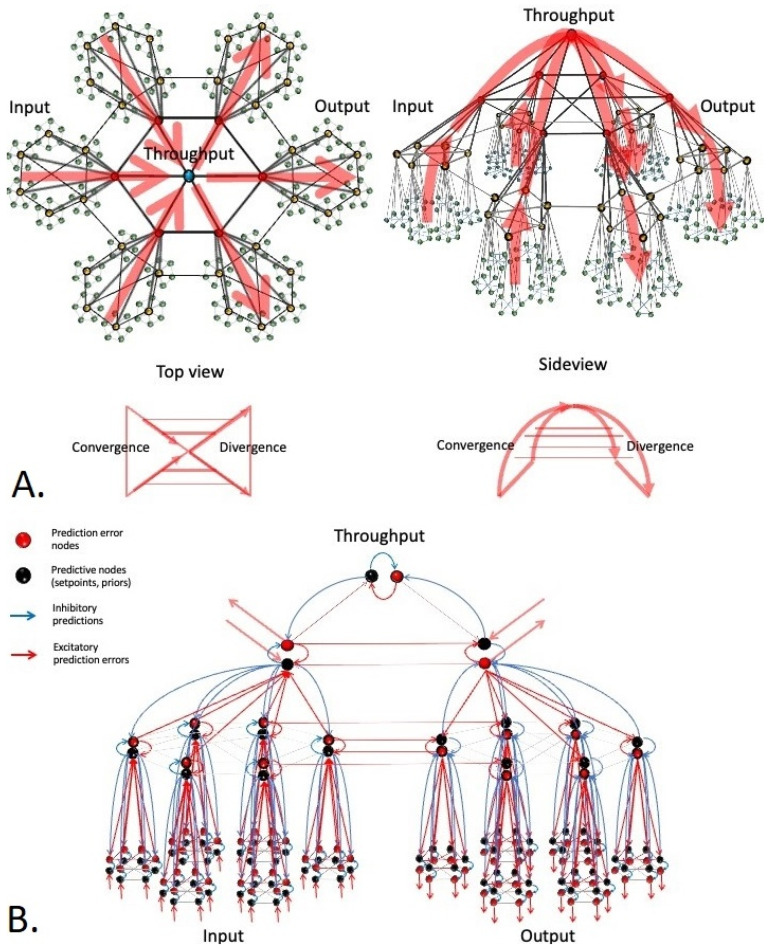
Consensus network structure that is proposed to support the process of active inference in all living systems. *Organisms can be conceived of as dual hierarchical Bayesian control systems that consist of an input hierarchy (left side), throughput hierarchy (top of the pyramidical structure), and output hierarchy (right side). Hierarchical message passing through these structures is thought to underlie hierarchical Bayesian inference in living systems. (**Panel A**). The structure shown in this figure integrates current ideas on hierarchical predictive coding [61,62] *with key findings from network science* [56,59] *and systems biology* [60,65]. (**Panel B**). This panel shows a cutout of the structure shown in panel A for closer inspection. Black nodes: priors (setpoints or predictions), red nodes: prediction error units. Blue arrows: inhibitory predictions, red arrows: excitatory prediction errors. Hierarchy of black nodes: a goal hierarchy (encoding world models). Hierarchy of red nodes: a hierarchy of evidence. At the base of the input hierarchy, input is compared to predictions (priors), and the residual error is projected upward in the hierarchy, where it is compared to higher-level priors (world models), and the process repeats. Prediction errors at some level of organization are used to both update priors (‘belief updating’) and inspire action. Predictions suppress prediction errors (‘explain away the evidence’). Note that prediction errors are escalated upward in the input hierarchy to update the goal hierarchy and downward in the output hierarchy to inspire action (**panel A**, top image, large red arrows). Predictions follow the opposite path to form a global counterstream,* i.e., *they are escalated upward in the output hierarchy and downward in the input hierarchy (not shown, but see **panel B**, small blue arrows). The entire structure has an information bottleneck or ‘bowtie’ structure, in which information (prediction errors and predictions) reaches maximum compression within the throughput hierarchy and is less compressed in input and output hierarchies (**panel A**). Note that local flows of prediction errors and predictions may deviate from the global flows (left to right, or right to left),* i.e., *counterflows may exist locally. Skip connections (horizontal red lines) allow for shortcuts between input and output hierarchies* e.g., *corticocortical connections), causing the bowtie to fold back onto itself (panel A, lower part)*.

In a recent paper [21], we proposed a consensus view on the ‘plumbing’ that makes active inference possible. The approach taken involves combining current knowledge on the structure of living systems with recent insights into their function. First, we show that all living systems follow the same architectural principles, i.e., they are *small world* network systems with a nested modular structure [56]. These are networks in which most elements (nodes) have few connections, but some have many. These highly connected units (hubs) ensure that the network as a whole has a small average ‘pathlength’, which is the average distance between any two nodes in the network when moving along the shortest paths. This causes signal transmission across small world networks to be highly efficient even in very large networks (e.g., in social networks, only six degrees of separation lie in between any two people on this world, making it ‘a small world after all’). Hubs contract parts of the network into so-called communities or clusters [57]. Such clusters may themselves serve as nodes at a higher spatial scale level of observation and so on. For example, organelles form cells that are a part of larger modules (tissues), which in turn are a part of supermodules (organs), etcetera, until one reaches the level of the organism itself. Thus, a hierarchy of part–whole relationships is formed (a ‘mereology’), in which one scale level of biological organization cannot exist without the other (e.g., [58]). The topological structure of such networks is the same across scale levels, which is why such networks are called scale-invariant or ‘scale-free’ [59,60]. We then show that all organisms appear to follow the same principles of network function (internal signal transduction, dynamics). This involves a combination of hierarchical message passing and predictive coding that has seen diverse representations and for which a consensus view has been proposed by Karl Friston [61,62]. In this view, all living systems are involved in some form of hierarchical Bayesian inference, i.e., modeling the latent (hidden) common causes behind observed events in their inner and outer worlds and updating these models using new evidence. In order to accomplish this, organisms have nodes that function either as prior (prediction) units or as prediction error units (Figure 5). Whereas prior units encode some predictive model of the world, prediction error units encode the difference between the model and newly obtained evidence. Such evidence initially enters at the bottom of the hierarchy in the form of excitatory input from the senses (bottom left in Figure 5). These input signals update the values of prior units, which in turn suppress the activity of prediction error units at the same hierarchical level by means of inhibitory connections. These prediction error units then try to update the values of prior units by means of excitatory connections, producing circularly causal dynamics (within-level oscillations). Since the suppression of prediction error by (updated) priors is rarely complete, a residual prediction error is produced that projects upward in the hierarchy to update the values of prior units at a higher level within the hierarchy. These units in turn project backward to suppress the same lower-level prediction error units by means of inhibitory connections, again producing circularly causal dynamics (between-level oscillations). Thus, each hierarchical level is involved in suppressing prediction error within that same level, as well as at a lower level. As observed above, the process of updating the values of priors by means of prediction errors is called ‘(Bayesian) belief updating’. The suppression of prediction errors by updated predictions is often referred to as ‘evidence’ that is ‘explained away’ by hierarchical Bayesian ‘beliefs’ [42]. Typically, prediction errors are fed forward until they are suppressed by a model of sufficient hierarchical depth, which is the model that best ‘explains the observed evidence’. Note that only prediction errors are carried forward through the hierarchy and not the original input from the senses. Quite fundamentally, this means that organisms have no direct access to the external world, from which they are separated by a barrier. What they perceive is a hierarchical model of the world that best explains the observed evidence, rather than a direct representation of the world [51,63].

The above dynamics is thought to underlie hierarchical Bayesian inference in living systems [61,62,63]. When applying this principle to scale-free network structures, one can see that the process of generating and updating Bayesian beliefs occurs at all spatial scale levels of organization within the nested modular hierarchy. Each scale level has an ‘input part’ (a collection of prediction error units) that connects to a higher-level ‘throughput part’ (a smaller number of priors that try to suppress prediction error), after which the residual error is fed back down the hierarchy to an ‘output part’ (a larger number of prediction error units), to produce output sequences. Crucially, the various priors and prediction error units in this configuration may involve network nodes or clusters, depending on the spatial scale level of observation. Thus, a self-similar (scale free/fractal-like) network structure is obtained in which the same input–throughput–output motif (a ‘feed-forward loop’ [64]) can be observed at each spatial scale level of observation: from the smallest scale of only three nodes (e.g., a neural circuit within the visual cortex) to a global ‘hierarchical Bayesian control system’ comprising the global compartments of perception, goal setting, and action control, which constitutes the organism (Figure 5). At each level of observation, prediction errors converge while ascending in the input hierarchy and diverge while descending in the output hierarchy, giving the structure the overall shape of a dual hierarchical (nested modular) ‘bowtie’ network structure [60,65]. Note that predictions converge while ascending in the output hierarchy and diverge while descending in the input hierarchy, to form a global counterflow. Information flows can take shortcuts via skip-connections that run between input and output hierarchies at comparable levels within the hierarchy, effectively causing the bowtie structure to fold back onto itself (Figure 5).

In forming hierarchical Bayesian models, organisms need to solve the binding problem [66], i.e., they need to figure out whether a set of events that occur simultaneously share a single common cause that should be encoded by a single variable (e.g., by a single network node or cluster), or whether these events represent separate causes that should be encoded separately (e.g., by separate nodes or clusters). In solving the binding problem, an important role is played by highly connected elements in these networks (so-called ‘hubs’). A hub can be pictured as a horse cart driver that needs to keep a team of horses in check, while using the reins to appreciate the general state of the team of horses as a whole (another example would be a middle manager that tries to get a sense of the general state of a team of employees). Every single horse keeps in touch with a part of the external world, but the driver itself tries to form a picture of the whole situation. This driver can in turn be seen as a horse that, together with other drivers, is kept in check by yet other drivers (directors), etc. The highest drivers (CEOs) thus try to get a sense of the global state of most horses in the hierarchy, through which they encode the most contextually integrated model of the experienced world, but only in a very compact and abstract sense. Similarly, living systems contain hub structures that converge onto hubs to form a nested modular network structure (a pyramidal shape), which encodes an increasingly integrated model of the world (Figure 5). Such nested modular collections of hubs are called ‘rich clubs’, since they are ‘rich in connections’ [67,68]. In Figure 5, a hierarchy of priors (black nodes) can be discerned that starts with the simplest of setpoints at the base of the hierarchy, to eventually involve only a few hub clusters at the top. Each subsequent level within this hierarchy encodes the hidden common causes behind a multitude of subordinate events using an increasingly small number of independent variables (degrees of freedom). Such integration takes place across multiple contextual cues in space (e.g., multiple horses influence the hub-driver at the same time), as well as time (e.g., the same horses show faster and slower dynamics, which are encoded vertically in the hierarchy) [69,70]. In other words, each subsequent level in the hierarchy encodes increasingly long-term predictions of increasingly complex econiches in an increasingly abstract and parsimonious way. Organisms, therefore, try to model their inner and outer environments using a shrinking number of variables but with minimal loss of information, meaning that some form of compression takes place while moving upward in the hierarchy [71]. In mathematical terms, information is funneled through an increasingly low-dimensional manifold (which has been compared to Occam’s razor) [72]. The apex of the pyramid shown in Figure 5 (the ‘knot’ of the bowtie), therefore, serves as an ‘information bottleneck’ structure [73] that encodes econiches at the highest level of ‘sophistication’ that an organism can achieve [65,74,75]. The term sophistication is used on purpose here, since it has been proposed to refer to predictive models that are models of other models (i.e. recursive beliefs: having beliefs about beliefs) [75]. In nested modular network systems such as Figure 5, higher hierarchical levels integrate across a range of lower levels (by means of hub nodes). Such integration takes place across multiple contextual cues in space, as well as time, causing higher-level models to encode increasingly long-term predictions of increasingly complex econiches in increasingly parsimonious (and abstract) ways. In other words, information bottleneck structures are used by living systems to build hierarchical Bayesian models using a minimum number of parameters (i.e., while minimizing model complexity costs). For this reason, we prefer not to call higher-level models more ‘complex’, since that term is reserved for models with many parameters. Higher levels do convey more long-term, abstract, and symbolic representations (i.e., a joint probability distribution over a set of prior probabilities under a hierarchical model [76]). This causes higher hierarchical levels to be relatively disconnected from events at lower levels, i.e., they encode models that model latent causal structure behind lower-level events with some degree of autonomy and creativity. Such ‘hierarchical generative models’ are able to escape the limitations of scarce and noisy data samples and nonetheless reach high levels of predictive accuracy, e.g., [77]. In living systems, the highest hierarchical levels encode contextually rich econiches that are to be explored or rather avoided in the near or further future [40]. Another way to refer to such hierarchical predictive models of econiches is a ‘goal hierarchy’ [20,78]. Goal hierarchies encode the logical set of econiches (goals) and corresponding subniches (subgoals) that the organism needs to pursue in order to reach the global econiche (goal) encoded at the top of the hierarchy [72].

As mentioned, prediction errors with respect to goal hierarchies serve not only to update these hierarchies and produce optimally informed models of the world, but also to inspire action [37,42,51]. Hierarchical Bayesian control systems are dual-aspect hierarchies in which the input hierarchy continuously supplies the output hierarchy with residual prediction error to coordinate behavior. When a simple goal state at some hierarchical level of inference and corresponding policy is insufficient to explain the evidence, the residual error is passed onto a higher level within the goal hierarchy, where a more sophisticated world model (goal state) tries to suppress prediction error. Any residual error then crosses over to corresponding levels of the output hierarchy to produce action sequences of corresponding levels of sophistication. Thus, a hierarchy of red hub nodes can be observed in Figure 5 that encodes a hierarchy of evidence, which is contrasted with the hierarchy of priors within the goal hierarchy to produce prediction errors at matching hierarchical levels that are fed into the output hierarchy to induce behavioral responses of corresponding levels of sophistication. Such output then serves to change the environment and produce a different niche model fit [37,42,51]. A common example is walking: this (habitual) motor pattern can in itself be sufficient to solve the problem of getting to a food source without much effort. When the terrain becomes rough, however, the organism may encounter obstacles that lie between itself and its goal (the food source). Such encounters produce prediction errors, which ascend in the hierarchy until they are suppressed by a sufficiently sophisticated model of the econiche (goal state). Prediction errors relative to this goal state then induce behavioral policies at a higher level of sophistication. For instance, the organism will now reorient itself (sample the environment to infer a model that encodes a richer environmental context) and plan a detour. Thus, goals and corresponding subgoals are pursued in a logical order by means of matching action sequences until the organism reaches its preferential global econiche [79]. Organisms can, therefore, be seen as hierarchical problem-solving machines that infer ever more sophisticated goal states and corresponding action–perception sequences until prediction errors are suppressed and the problem is solved. Since the level of sophistication of each behavioral response matches the sophistication of its corresponding goal state, which in turn matches the organism’s optimal perceptive model of the world, organisms automatically produce ‘adaptive’ behavior that is flexibly tuned to fit the level of complexity of their actual environments [21,80].

Interestingly, the output hierarchy is also involved in some form of inference [80,81]. In output hierarchies, the sensory states of output organs (such as muscles or endocrine glands) are used to model the actual actions that are taking place, whereas prediction errors with respect to such models are used as output signals to these organs to make on-the-fly corrections (Figure 5). Thus, hierarchical Bayesian control systems have input hierarchies that try to figure out “what the world is doing” (perception), output hierarchies that try to infer “what the organism is doing” (action control), and throughput hierarchies that try to infer “what the organisms *should* be doing” (goal setting) [21]. These domains enter in a closed (feedforward-feedback) loop with the environment to allow for active inference.

## 6. How Information Processing in Living Systems Corresponds to Behavior

In order to understand how stress alters the behavior of organisms in a universal way, we need to understand how message passing at different levels within hierarchical Bayesian control systems correspond to different forms of behavior. In this view, the lowest levels within such systems produce basic stimulus–response patterns called reflexes (e.g., sweating or salivation or spinal reflexes such as locomotion). In control theory, low-level reflex arcs such as these are said to produce ‘homeostatic’ reflexes, i.e., the closest regulators of a low-energy stable state (homeostasis) [19]. When moving upward in the regulatory hierarchy, more sophisticated action–perception cycles are formed that consists of combinations of basic reflexes, e.g., fighting, fleeing, freezing, feeding, reproducing, resting, digesting, self-repairing, and (parental) caring in response to typical cues. Such complex reflexes are called instinct patterns in evolutionary psychology [82]. When moving further upward in the regulatory hierarchy, more sophisticated policies are formed, which are called ‘habits’ [83]. These are automated responses to typical stimuli that consists of a combination of reflexes and instinct patterns in response to more complex perceptual cues (e.g., taking a morning stroll involves combination of reflexes and instinct patterns such as walking, resting, and digesting). Lastly, the highest levels of the regulatory hierarchy produce ‘goal-directed’ behavior, which involves nonautomatic (i.e., effortful) actions based on explicit and often long-term predictions of the consequences (perceptual outcomes) of actions [84]. Such predictions take the form of ‘simulations’ of what might happen if some action is taken. The predicted outcome of certain actions is then a prerequisite for such actions to be selected as the policies that are most likely to suppress prediction errors across trials [20,80]. In control theory, goal-directed behavior is considered a form of ‘allostatic’ behavior, i.e., behavior that is produced by hierarchically higher regulators that are superposed onto lower-level regulators in order to secure stability by means of more sophisticated responses when lower levels and less sophisticated forms of behavior fail to do so (i.e., “stability through change”) [19].

Together, these different forms of behavior develop over the course of many iterations of trial and learning from prediction error (active inference). In this context, learning refers to a process of Bayesian belief updating, where prior expectations are updated in response to novel evidence (prediction error). Such updating involves a change in the efficiency (or complete rewiring) of the connections between priors, which corresponds to the actual learning process [43]. Belief updating may occur at any level within the hierarchy of priors shown in Figure 5. At the lowest (reflexive) levels of the hierarchy, belief updating produces a form of associative (stimulus–stimulus) learning that is called ‘Pavlovian learning’ (classic conditioning). During Pavlovian learning, organisms gradually associate one (familiar) stimulus with a new one and produce the same behavior to either of these stimuli (e.g., dogs learn to associate the ringing of a bell with food, causing anticipatory salivation). Belief updating at ‘intermediate’ and ‘higher’ levels within the hierarchy of priors is referred to as ‘habit learning’ and ‘goal-directed learning’, respectively. Pavlovian learning and habit learning have been observed in a wide variety of species, including bacteria. Although goal-directed learning is usually associated with ‘higher’ species, many aspects of behavior in ‘lower’ species (including bacteria) resemble goal-directed behavior [84]. This means that similar forms of learning and behavior are present to different degrees in different species, depending on the sophistication of their goal hierarchies. Similarly, within-species individual differences in inferential abilities and behavior are thought to be due to differences in the outgrowth (maturation) of goal hierarchies during the lifetime of the organism. The next paragraph examines what types of world models are encoded at the top of goal hierarchies and to what kind of behavior they give rise. After that, we examine how changes in hierarchical Bayesian control systems correspond to shifts in behavioral policies under rising levels of stress.

Organisms are known to construct at least two distinct types of world (econiche) models at the top of their goal hierarchies: models of their external environments and models of their internal environments [85]. Such models inspire behavior that purports some sense of agency, i.e., the ability to distinguish between events that are generated by the organism itself versus events that have their origin outside of the organism [86,87]. The former include signals that arise within the body of the organism, as well as signals out of the body that have been produced by the organism itself, such as sounds or vibrations due to its own movement [86]. Basic forms of self (versus non-self) encoding have been observed even at the level of bacteria and may take more elaborate forms in higher mammals [88]. Such models increase in contextual richness when they gain in complexity and hierarchical depth, which appears to underlie the distinction between ‘higher’ and ‘lower’ species [37,51]. Self-models may include any form of self-representation, such as a body image and a psychological self-image [89]. Such models encode self-referential (personal) goals that the organisms would like to occupy or sample. Prediction errors with respect to such global goals inspire behavior that is aimed at achieving these goals through a logical series of subgoals and corresponding behavioral policies [72]. For instance, the global goal of catching food requires the global policy of hunting, which consists of subpolicies such as hiding, freezing, fighting, and eating. Reaching such goals involves the mastery of personal skills that vary from hunting and gathering and building nests to finding shelter and mastering survival skills (or occupational skills in humans). The growing mastery of such skills is referred to as self-actualization or the development of agency [87,90,91,92,93]. Especially in higher social mammals, models of the external world include social models (‘theories of mind’) [92,94]. Such models try to infer the hidden common causes behind multiple signals in the external world that are produced by other organisms, i.e., the intentions and motives of friends, rivals, mates, or kin [92]. Prediction errors relative to such models inspire behavior that is aimed at achieving personal or interpersonal (social) goals by taking these motives into account. Such actions may involve e.g., offensive or defensive actions, courtship rituals, parental investment, or nursing behavior. The increasing mastery of social skills is called social learning [95]. Note that even some forms of antisocial behavior (e.g., deceit or fraud) require the presence of social models, since such behavior requires some degree of knowledge of the intentions of others, which is used to one’s own advantage. Regardless of the type of species, self-models and social models involve more integrative (goal-directed) forms of inference that occur at higher levels within a goal hierarchy (see previous section and Figure 5). In our recent paper [21], we showed that external (social) models are likely to form the top of the input hierarchy, since these are involved in inferring ‘what the outside world is doing’. Following the same line of reasoning, internal (self) models are thought form the top of the output hierarchy, since these are involved in inferring ‘what the organism is doing’. These assumptions are confirmed in the human brain [21], but require confirmation in other species. Since the timescale of events is encoded vertically in hierarchical networks [69,70], the vertical outgrowth of self and social models allows organisms to incorporate increasingly long-term predictions with respect to increasingly abstract personal or interpersonal goals. For instance, self-models and social models in higher primates have reached a level of sophistication that allows them to imagine and work toward complex social positions across many years of time.

**Figure 6:**
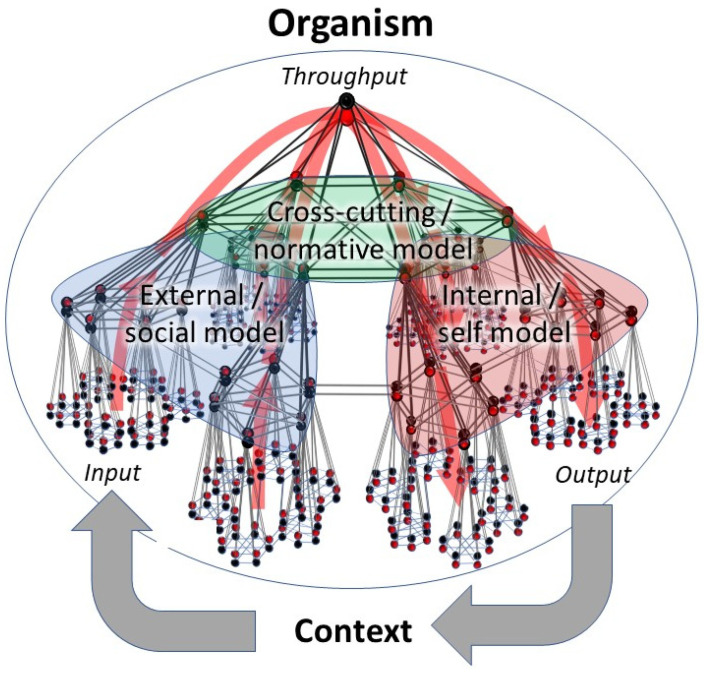
The putative positions of different world models in living systems.

For a long time, it was thought that organisms only construct these two global models, i.e., internal (self) models and external (social) models. In our recent paper [21], we demonstrated that the principle of hierarchical Bayesian inference logically (and necessarily) dictates that there must exist a third, highest level of inference, whose job it is to infer the hidden common causes behind events that involve both the internal and the external world of the organism, across multiple context factors in both space and time. In short, there must be an overarching model that integrates across self and social models to encode a commonly held world model (a common econiche) (Figure 6). Prediction errors relative to such models inspire actions that are aimed at affecting this common econiche rather than the local, internal, or external (social) niches of the organism itself. Although in theory, knowledge of a ‘common ground’ can be used solely to the advantage of an individual organism or local group, such knowledge is unlikely to produce strictly selfish policies since any type of behavior that favors a global goal (i.e., promotes global stability) eventually also favors individual organisms and local groups (i.e., promote local stability). Especially in higher social species, the vertical outgrowth of overarching models allows organisms to produce increasingly sophisticated models of common econiches across increasingly lengthy periods of time. Prediction errors relative to such models inspire behavior that is aimed at promoting long-term collective stability, such as an equal sharing of energy and resources across multiple stakeholders (e.g., collaboration, food sharing, and other forms of distributive justice), resolving conflict situations (e.g., mediation or arbitration), or holding each other responsible when goals are violated that apply to all members of the community (punishment for norm violation and other forms of justice). Normative or law-abiding behavior of this kind (including altruistic behavior) has been observed in some form or another in a wide range of organisms, from unicellular organisms and invertebrates to higher vertebrates and mammals [96,97,98,99,100]. Whereas a clear self–other dichotomy seems to mark the distinction between kinship selection (i.e., the favoring of kin over others, nepotism) and reciprocal altruism (i.e., investing in unknown individuals) [101], the hierarchical expansion of overarching world models seems to soften the self–other dichotomy by pushing behavior toward an increasingly inclusive (social) space and toward ever larger (transgenerational) timescales, i.e., devoting time and energy to improve the stability of unknown future individuals and species [102,103,104,105,106,107]. Such overarching world models allow organisms to escape the polarization or nepotism that is inherent to local self-referential or interpersonal goals by appealing to commonly held niche models that are invariant across generations. Especially in social organisms where regulatory hierarchies have reached high levels of sophistication, such shared setpoints may take the form of community norms or values [106,107,108,109,110]. Such goals promote social cohesion between large numbers of individuals across substantial individual differences and substantial spatial and temporal boundaries [111]. Even the ability to see all of life as connected under such common laws and insights (which includes religious insights and corresponding feelings) may be caused by this highest level of inference (e.g., [112]). In this respect, it is interesting to note that ‘religare’ originally means ‘to reconnect’ in Latin (across individual differences and timeframes, under a common highest law), that Catholicism means ‘(moving) toward a whole’, Islam means ‘order/peace through submission (to a higher law)’, and ‘hierarchy’ refers to ‘holy ordination’ in ancient Greek. In short, organisms are likely to be engaged in a highest level of inference at the top of their goal hierarchies, which tries to infer what the organism “should be doing”. Such overarching (normative) world models are not restricted to higher organisms, although organisms with more sophisticated goal hierarchies do tend to show more sophisticated forms of behavior (see previous section for a definition).

## 7. Disorder: A Collapse of Hierarchical Control

We now turn to the point of explaining the apparently universal stress response of organisms in terms of the actions of hierarchical Bayesian control systems, as laid down in the previous sections. To summarize, this generic response is composed of the following elements: as a first rule, rising levels of stress produce characteristic changes in internal message passing of living systems. These involve an increase in the strength of (auto)correlations and variance observed between the various components of a living system. This happens up to a discrete ‘tipping point’ (or bifurcation), after which (auto)correlations drop but variance remains high. Such changes are captured by a single variable of permutation entropy, which shows that the dynamics of signal transduction within organisms turns increasingly disorderly until a tipping point is reached (Figure 1). Such changes coincide with the phenomenon of critical slowing down (CSD): a delayed recovery after perturbation of the system. When systems move beyond the tipping point, correlations decrease but variance and entropy levels remain high until the system fails completely. As a second rule, the timeseries of overt behavior of organisms follows the same pattern as internal signal transduction: disorder levels gradually rise until a tipping point is reached. Thirdly, rising levels of stress change the content of an organism’s behavior in an apparently universal way: low levels of stress induce routine (reflexive or habitual) behavioral policies, whereas moderately high levels cause organisms to show more sophisticated (goal-directed) forms of behavior. When exposed to extreme (near-lethal) levels of stress, behavior shifts from ‘slow’ to ‘fast’ behavioral policies [14], i.e., organisms shift their focus from a long-term commitment to fellow organisms and reproductive activity to behavior that is focused largely on the preservation of self and/or kin. This corresponds to a shift back from goal-directed to habitual forms of behavior. Lastly, when living systems remain challenged after having passed the tipping point, they willfully disintegrate (i.e., lose their independence from the environment). The state of the system will now linearly follow that of the environment, amounting to a loss of homeostasis (i.e., an unstable, high entropy state). Such tipping points usually correspond to malfunction, disease, or the death of a system.

The sum of these observations can be explained by looking at the actions of hierarchical Bayesian controls systems, as shown in Figure 5. We argue that ‘prediction error’ can be read as ‘stress’ and ‘action’ can be read as the ‘stress response’, such that the theory of active inference can be applied to stress research [21]. In this view, any change in environmental conditions may alter an organism’s perception of the world, which produces a different fit with the organism’s predictive models of the world (goal states). This prediction error (‘stress’) is used to adjust the predictive model (i.e., belief updating, learning) and converted into action (a stress response). Hence, when we feel stressed, we actually perceive the mental and bodily changes that constitute a stress response to a prediction error. Incidentally, this means that stress can be reduced in two fundamentally different ways: either by performing an action or by changing expectations or beliefs. This view has been highly influential in the psychological literature and is applied worldwide, for instance, during cognitive behavioral therapy (CBT) [113,114].

As mentioned in Section 5, the ascent of prediction error in goal hierarchies adds levels to a hierarchical model of the world up to a level of sophistication that sufficiently explains the observed effects. Prediction errors relative to this model are then used to inspire behavior of corresponding levels of sophistication, starting from simple, low-level reflexive (e.g., walking) or instinctive forms of behavior (e.g., foraging) to habitual (e.g., take a morning stroll) and goal-directed forms of behavior (e.g., finding the shortest route to a food source in a complex environment). When prediction error (stress) ascends in information bottleneck structures such as Figure 5, this causes an increasingly large number of lower-level systems (horses) to be ‘enslaved’ by an increasingly small number of high-level hub regions (drivers). Rising levels of prediction error, therefore, initially increase the amount of centrally coherent governance (top-down hierarchical control), causing the subordinate systems to become increasingly synchronized (coherent). Thus, we propose that the observed increase in correlations between the various components of systems that are stressed in the mild-to-moderate range is due to an increase in central governance exerted by high-level hub structures (Figure 5). Similarly, we propose that the observed increase in the total variance of such systems may be due to the recruitment of increasing numbers of subordinate systems. This is because each of these subsystem produces its own within-level and between-level oscillations between prediction and prediction error units, which correspond to unique amplitudes and variances (frequencies). Since the increased involvement of hub structures raises the connectivity between system components, the number of recurrent connections between such components is also likely to rise. Subsystems will, therefore, increasingly reinforce each other’s activity through circularly causal connections to the point where it takes longer for stressed systems to recover from initial perturbation. This may explain the phenomenon of hysteresis or ‘slowing down’, as quantified by rising autocorrelations (see Section 1). Together, these changes are likely to affect the permutation entropy of the system (Box 1). On the one hand, the increase in central integrative governance exerted by hub structures synchronizes signal transduction between lower-level (subordinate) domains, which imposes some degree of order and decreases the permutation entropy of the system. On the other hand, however, every level that is added to a hierarchical model increases the number of microstates (and microvariances) required to describe the total state and evolution of the system. Since the amount of information required to describe the total state and evolution of a system is equal to its (weighted) permutation entropy [23], the recruitment of additional systems will raise entropy scores. Thus, an equilibrium will ensue between ‘order through synchronization’ by hub units and ‘disorder through recruitment of additional subsystems’. This balance may at times favor either order or disorder at different trajectories within the mild-to-moderate range, but empirical studies show that rising levels of stress eventually cause a *net* rise in permutation entropy levels (see Section 1).

**Figure 7:**
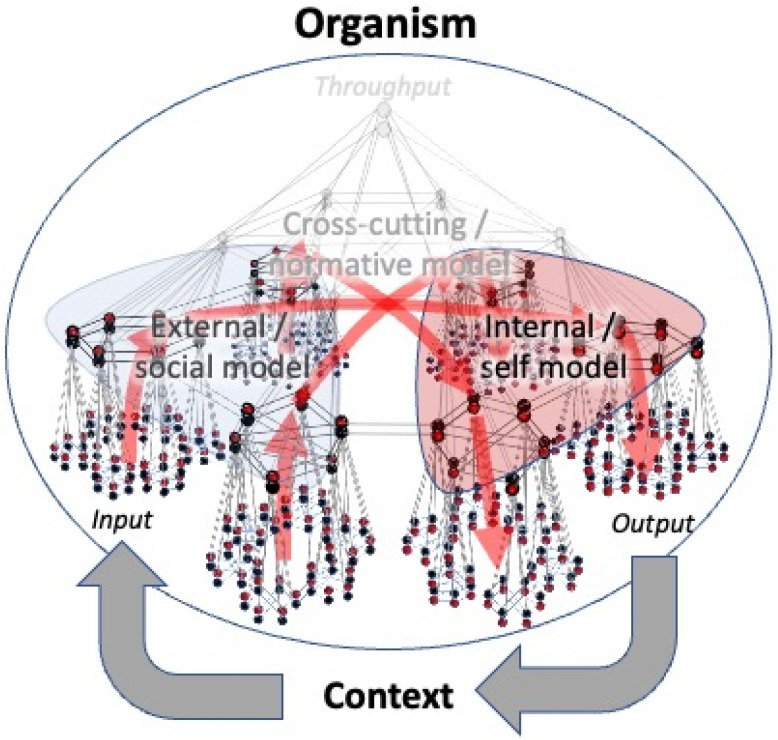
The top-down collapse of a goal hierarchy under severe levels of stress. *The vertical escalation of free energy (model error) in bowtie network structures causes hub structures within the top of the hierarchical pyramid (the knot of the folded bowtie) to overload and fail as a function of node degree (the number of connections per node). Since such hubs maintain global (functional) connectivity within the network structure, their failure causes a top-down collapse or ‘flattening’ of hierarchical structure (i.e., a loss of nested modularity). Prediction errors (large red arrows) and predictions (not shown) then seek the shortest path from input to output (or vice versa) via horizontal skip connections, effectively ‘bypassing’ integrative processing at higher (allostatic) areas within the hierarchy, to produce less well-informed (homeostatic) forms of behavior. This is a biophysical model of ‘allostatic overload’, which is a dominant theory that explains physiological changes and shifts in behavioral policies in organisms under extremely stressful conditions. See text for further details*.

Although organisms can recruit ever higher (allostatic) levels of control to enhance the sophistication of their (stress) responses, this cannot go on indefinitely. Since any hierarchy is finite, there must be some limit to the modeling and problem-solving capabilities of an organism, i.e., some prediction errors cannot be suppressed even by the most sophisticated models an organism can produce. Such models are encoded at the top of the goal hierarchy (the knot of a bowtie), which contains some of the most central hub structures of the system. When prediction errors reach the top of a goal hierarchy, these high-level hub structures are continuously triggered by prediction errors (stimuli) that originate from any direction within the network structure. In order to respond to such excessive stimulation, hubs require more metabolic energy than they have access to. When energy demand exceeds energy supply, this causes hub units to congest and shut down: a phenomenon called ‘hub overload and failure’ [115]. This can be compared to a high-level horse cart driver that is overpowered by the sheer number of horses that need to be restrained. In biophysical terms, hub units reach the limits of their capacity to dissipate energy back into the environment. Studies show that the most connected nodes in a network (hubs) are most sensitive to such overload [116]. This means that high levels of stress cause a selective targeting of hub structures in living systems. Although *small world* network systems are known to be robust to random attacks of nodes and links, they are very sensitive to targeted attacks of hub nodes [117]. Since hub nodes maintain the global connectivity of living network systems, the selective targeting of such units will cause such systems to fall apart in a top-down fashion, as a function of node degree: the loss of only a few high-level hubs will cause information flows to be relayed to hub structures in subordinate parts of the network, which may subsequently get overloaded, etc., until the system is only capable of low-level performance (Figure 7). Cascading failures such as these have been described in power grids, transportation networks, and stock markets [118,119], as well as in biological systems [51,52] and social networks [120,121]. Since the most sophisticated models are produced at the top of a goal hierarchy, the top-down collapse of a regulatory hierarchy forces organisms to move from allostatic (more sophisticated and goal-directed) to homeostatic (less sophisticated and habitual) levels of control. To our knowledge, this is the first mechanistic account of the phenomenon of ‘allostatic overload’, which can be read as a process theory for shifts in behavioral policies toward ‘survival mode’ under severe levels of stress (e.g., [18]). It is important to note that this loss of hubs is initially of a functional nature, i.e., they become unresponsive to stimulation, but retain their structural connections, causing a loss of functional but not structural connectivity. When hub overload persists (i.e., when stress is chronic), hubs may become permanently unresponsive, causing a loss of structural connectivity and permanent damage to system integrity [122].

**Figure 8:**
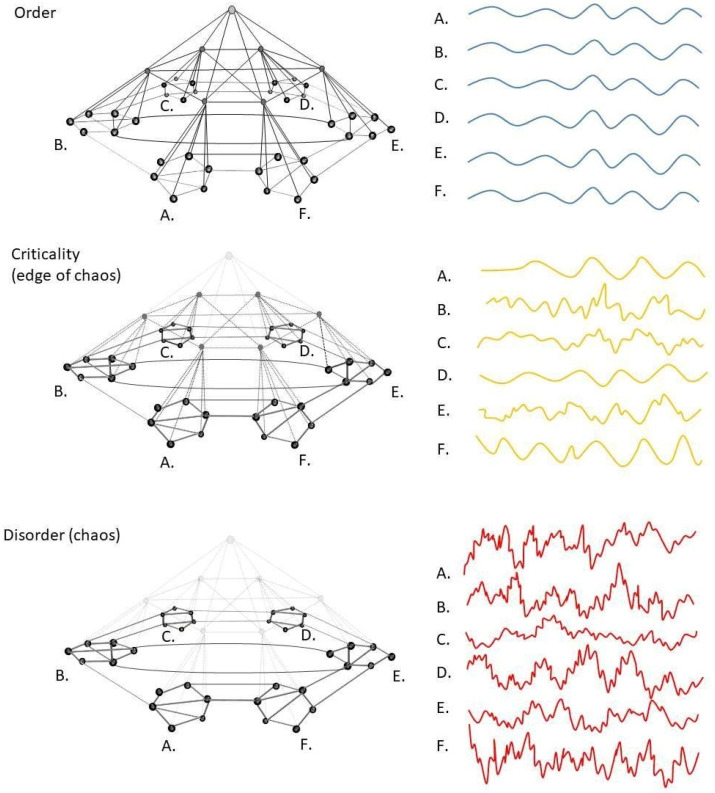
Explaining disorder and tipping points in stressed systems. *The balance between order and disorder of a system is at last partially controlled by the coupling of subsystems by central connectors (hub units). Disorder (including disease) may result from a loss of centralized and integrative coupling that is caused by a top-down collapse of hierarchical control systems, due to hub overload and failure. This causes a loss of coherence and increased variance at lower levels within the hierarchy that translate into increased levels of permutation entropy scores (see text). Organisms self-organize toward a dynamic state in between complete order (i.e., perfect synchrony of timeseries A–F; upper picture) and complete disorder (i.e., perfect randomness of timeseries A–F; lower picture). This equilibrium state is called self-organized criticality (SOC; ‘the edge of chaos’; middle picture). A loss of higher (integrative) hierarchical levels of control may shift this equilibrium toward the disordered side of the spectrum. Although stress levels may rise gradually, the loss of high-level central control by a cascading failure of high-level hubs structures is a discrete process, causing discrete transitions from order to disorder. A stress-induced loss of hub structures may, therefore, explain sudden phase transitions that mark the onset of physical or mental dysfunction, disease, or death. See text for further details*.

Cascading failures typically involve the occurrence of tipping points [123]. The abruptness of the change seems to be due to the fact that, at some critical point, only a small change (e.g., the overload of a single hub node) may be sufficient to cause a chain reaction that leads to the collapse of a large part of a hierarchy [118,119]. The collapse of goal hierarchies will leave subordinate structures of the network without central guidance, causing the balance between functional integration (order) and segregation (disorder) of states to tip over toward desynchronization and ‘disorder’ (e.g., the horses will panic and start running wild when the driver falls away) (Figure 2 and Figure 8). This may explain the sudden rise in permutation entropy that is universally observed in the timeseries of severely struggling systems. Hub overload and cascading failure may similarly explain the decrease in number and strength of correlations between system components in terms of the loss of central integrative connections (reins) maintained by hubs. In contrast, variance remains high since lower-level systems are no longer coupled and suppressed by higher-level priors, yet they are continuously excited by incoming prediction error. This overexcitation of subordinate systems is called ‘disinhibition’ in the psychological sciences [124]. The massive involvement of independently responding and disinhibited microstates is likely to make an important contribution to rising permutation entropy scores (see Section 1) [22].

Since failing systems are characterized by low levels of (auto)correlations and high levels of variance, this means that the amplitude-to-error (signal-to-noise) ratio of the system decreases. In active inference theory, the signal-to-noise ratio is called the ‘precision’ of the signal (i.e., a quantity that expresses the level of confidence that the information conferred by the signal is correct). Thus, allostatic overload is a process where model complexity costs are reduced at the expense of long-term precision (see [125] for a mathematical description of this tradeoff). This makes sense from an evolutionary perspective, where stressed organisms may become quick to respond but less precise in their actions, as long as this saves energy and resources. An advantage of this mechanism is that organisms will have to spend less time and energy on the integration of large amounts of complex information (i.e., a reduction in model complexity costs). Prediction errors can now pass from input to output areas across skip-connections while avoiding much processing in higher-level throughput areas (goal hierarchies) (Figure 7). This allows organisms to respond more quickly and strongly to certain situations (disinhibition), providing them with just the edge needed to escape from a dire situation. As a disadvantage, however, goal hierarchies may become so shallow and noisy (i.e., unsophisticated and imprecise) that the corresponding behavioral policies will lack hierarchical correspondence with the environment and fail to suppress prediction errors in an effective way. In other words, overly flattened goal hierarchies will produce ‘maladaptive’ behavior. Such inefficient problem solving will cause the system to require more time to quiet down after initial perturbation, which adds to the phenomenon of (critical) slowing down. In addition to changes in internal message passing (such as circular causal loops between system components that keep re-exciting the system as discussed above), critical slowing down can, therefore, be explained by an insufficient suppression of prediction error through maladaptive action.

In summary, we expect that low-to-moderate levels of stress produce a net shift of the balance between functional segregation and integration of message passing in living systems in favor of functional integration by hub structures, corresponding to a gradual rise in (auto)correlations, variance, and permutation entropy scores. When stress levels increase further, a tipping point is reached at which central coherence by hub structures is suddenly lost, causing a steep rise in permutation entropy scores. These conclusions are in line with experimental data that show how changes in network topology may contribute to the formation of tipping points [10]. Our model seems to explain several generic changes in internal message passing of living systems under rising levels of stress. The next paragraph focuses on changes in the overt behavior of struggling organisms.

As observed, the various form of behavior that are produced by an organism reflect the level of sophistication of its internal states. Changes that affect internal message passing of stressed organisms will, therefore, produce behavioral changes that can be observed externally. To explain the shift away from *slow* to *fast* behavioral policies in stressed organisms, we propose that the top-down collapse of goal hierarchies causes organisms to shift from high-level goal-directed (allostatic) to lower-level habitual or even reflexive (homeostatic) forms of behavior (see Section 5). Since high-level goal states are responsible for factoring in all kinds of context factors in both space and time (past, current, and future scenarios of increasing complexity), the collapse of such models will cause organisms to pursue less sophisticated and more short-term goals: a ‘decontextualization’ of behavior (see Section 6). Since the top of the goal hierarchy encodes world models at the highest levels of sophistication (i.e., contextual integration in both space and time), this may explain why long-term and socially inclusive (normative) goals are often the first to go. Organisms will instead move toward more short-term and socially selective forms of behavior, which may include a shift from transgenerational and reciprocal altruism toward kinship selection (‘nepotism’) and self-preservation, potentially at the cost of other organisms and kin (e.g., maternal cannibalism in rodents). In the words of Brecht, ‘Zuerst kommt das Fressen, dann kommt die Moral’ (fodder comes first, then comes morality). The collapse of normative goal states may sharpen the self–other dichotomy, which may manifest as increased ingroup–outgroup behavior (polarization). When stress persists, external (social) and internal (self) models may be next to collapse. When external models disintegrate, individuals will make less sophisticated models of the goals or intentions of others, for which reason behavior will appear to become increasingly asocial in nature. This means that even some forms of antisocial behavior (e.g., deceit) are likely to diminish, since these require some insight into the motives and intentions of others (see below). Behavioral signs of collapsing social goal hierarchies may include lesser amounts of (long term) kinship-promoting activities such as parental or grandparental investment. With the possible exception of (grand)parents that sacrifice themselves for their offspring and admirable individual differences, it can be stated that severe and prolonged stress levels will generally cause organisms to economize on long-term and socially inclusive policies to focus on self-preservation, to the point where even self-preservation is at stake. When internal (self) models disintegrate, this causes fragmented and aimless behavior. Together, such changes may translate into rising levels of permutation entropy in behavioral timeseries, including constituent elements such as decreased (auto)correlations and high levels of variance (see below). When goal hierarchies collapse further, the decoupling between system components may become so severe that the system as a whole disintegrates. The internal state of a system will then linearly follow that of its environment (i.e., a complete loss of homeostasis), which usually corresponds to disease or the death of the system. In short, the overload and cascading failure of central integrative control may explain several of the generic behavioral features of living systems under rising levels of stress.

## 8. Permutation Entropy as a Universal Disorder Criterion

In the previous section, we showed that living systems can be modeled as hierarchical Bayesian control systems in which central integrative (allostatic) control falls apart in a top-down manner as a result of rising levels of stress, which can be defined as prediction error or variational free energy. Given the multitude of observations that similar behavior can be observed in nonliving systems, one may wonder whether more general laws exist that underlie such changes in living and nonliving systems. In this paper, we argue for the latter position by showing that living systems are a special class of open dissipative systems, for which general rules apply. Open dissipative systems are collections of coupled nodes that receive a constant flux of energy or matter from their surroundings, which they need to get rid of (dissipate) in the most efficient way possible [126]. Experimental studies and in vivo experiments have shown that the most efficient way in which networks can dissipate energy back into their environments is when their nodes organize themselves into nested modular (hierarchical) structures [127] and start to oscillate [116]. Apparently, the short pathlength and nested modular structure of small world networks (e.g., living systems) result from the necessity to dissipate energy back into the environment as efficiently as possible. The same can be said for the emergence of oscillations, e.g., in gene activity, insulin secretion, neuronal firing rates, or social rhythms. The simple necessity for efficient energy dissipation apparently causes the spontaneous emergence of ordered patterns in both structure and function of coupled systems: a phenomenon called ‘self-organization’ [11,128,129]. As observed in Section 3, the *local* emergence of ordered patterns (e.g., crystals) is allowed as long as this leads to a *global* increase in the entropy of the universe. Similarly, living systems have found a way to temporarily maintain their local form and order, by being able to dissipate energy as efficiently as possible back into the environment (which is to reduce variational free energy). This means that living systems will lose their internal coherence and fall apart when free energy (stress) is not dissipated quickly enough into the environment. We argue that this is essentially what happens in any system that is loaded up with free energy (stress) beyond its capacity to dissipate it back to the environment: the accumulation of such energy will cause a disintegration of system components and system failure (i.e., malfunction and death), causing a rise in permutation entropy scores. This is explained further below.

In lifeless open dissipative systems, the flow of energy through a system is mediated by its components that engage in some form of coupling. For instance, granular media such as water molecules, snowflakes, grains of sand, or pieces of the Earth’s crust act as coupled components that distribute chemical or mechanical energy across a network of similar components [49]. As observed, the simple need for optimal energy dissipation causes such systems to self-organize toward a network structure with an optimal level of (nested) modularity [49]. Such structural characteristics are in turn thought to influence the dynamics of such systems, producing a dynamic interplay between the structure and function of the system [130]. Since the various scale levels of a nested modular network system correspond to different levels of segregation and integration of energy flows [127], this means that open dissipative systems automatically arrive at an optimal balance between the integration and segregation of energy flows. Whereas functional integration corresponds to some degree of predictability through synchronization (order), functional segregation corresponds to a state of relative randomness through desynchronization (chaos). Thus, systems of coupled oscillators self-organize toward an equilibrium state in between order and chaos that is called ‘self-organized criticality’ (SOC) [131]. This ‘*edge of chaos*’ [132,133] is a special place where the level of coupling between system components is such that energy flows are able to propagate through the network with enough freedom to cause ‘cascades’ of node activity of some size and duration before dying out; too much coupling will cause such cascades to die out quickly (when coupling is inhibitive) or rather produce massive synchronization (when excitatory); both phenomena involve a state of high predictability or ‘order’. Too little coupling, on the other hand, will cause a lack of synchronization and ‘disorder’. Studies show that the transitional zone between ordered and disordered states of network systems is a discrete one, i.e., such zones are referred to as ‘phase transitions’ or ‘tipping points’ (‘bifurcations’, ‘catastrophes’, ‘percolation points’, or ‘regime shifts’) [123]. Tipping points describe a situation where only a small amount of energy is sufficient to push a system from one global (integrated, ordered) state into another (segregated, disordered) state [123]. Examples of such states in nonliving systems are melting or boiling points, where, e.g., ice represents a highly ordered state with strong connections between water molecules and only a small increase in temperature is sufficient to cause a cascading failure of hydrogen bonds (i.e., melting), allowing all water molecules to move around more freely as water. The exact origins of tipping points are still unknown, but network topology appears to be an important factor [10,130]. In systems of coupled oscillators, the flow of energy may arrange system components in such a way that it will arrive at a point where only a few central nodes are responsible for connecting all of the system’s nodes into one giant ‘connected component’ [11]. The removal of only a few of such nodes due to energy overload may then trigger a cascading failure [119], causing the system to lose global connectivity and move from a state of relative order to a state of relative disorder. Such transitions may occur in any (randomly wired) open dissipative network system, but are especially prevalent in nonrandomly wired (‘nonegalitarian’) systems where a few key connectors (hubs) are responsible for maintaining global connectivity [134] (Figure 8). Since living systems in most cases tend to be of the nested modular and nonegalitarian type [135], this may explain why critical phenomena are frequently observed in struggling organisms. We believe that the nonegalitarian nature of living systems has been insufficiently incorporated in today’s models of tipping points or critical slowing down, and that doing this may significantly improve those models.

In living systems, information processing takes the form of hierarchical Bayesian inference, which can be equated to free-energy dissipation in nested modular systems (a gradient descent on free energy, see above). The need for efficient energy dissipation (information processing) will cause living systems to automatically tune toward a level of nested modularity and corresponding equilibrium between integration (order) and segregation (chaos) that allows for optimal message passing. This means that the *edge of chaos* is a place where conditions for hierarchical Bayesian inference are optimal: too much coupling (functional integration, order) will interfere with the articulation of hidden causal factors (and, hence, model formation), but so does too little coupling (functional segregation, disorder). Instead, organisms automatically produce world models of optimal hierarchical depth (sophistication, see above). A simple need to get rid of an excess of free energy will cause living systems to automatically tune toward a point where information processing is optimal and (consequently) where the stress adaptation mechanism of organisms can operate most effectively. In other words, the basic laws of thermodynamics appear to cause living systems to automatically produce adaptive behavior in response to environmental fluctuations, to the best of their abilities. Of course, this is only true up to a certain (tipping) point. When the influx of free energy (stress) exceeds the dissipation capacity of the organism, a point will be reached where only a few key connectors are responsible for maintaining global network connectivity. At that point, even a small increase in free-energy levels (stress) will cause such structures to shut down, triggering a cascade that causes to the system to fall apart into disconnected components. This pushes system dynamics over the edge of chaos, toward disorder and system failure (Figure 8). The overflattening of a goal hierarchy therefore produces Bayesian models of suboptimal sophistication that cause the organisms to show maladaptive behavior (i.e., dysfunction or disease; see below).

This concludes our discussion of the emergence of disorder in living systems under conditions of severe stress. We showed that severe stress can be defined as an influx of (free) energy beyond the capacities of open systems to dissipate energy back to the environment. This causes a selective targeting of hub structures that maintain a nested modular hierarchy. The subsequent collapse of hierarchical structure involves a transition from a relatively ordered (synchronized, integrated, adaptive) state to a relatively disordered (desynchronized, segregated, maladaptive) state. The top-down collapse of goal hierarchies in living systems appears to be a special case of cascading failure in open dissipative systems that overload with free energy. Losing control and the sudden emergence of disorder may, therefore, be a universal feature of any open system that disintegrates as a result of a free-energy overload. As a result, permutation entropy (or any other suitable measure of disorder for that matter) may serve as a universal disorder criterion.

## 9. Disorder as a Universal Measure of Disease

In living systems, the term ‘disorder’ is often used as a way to describe dysfunction or disease of such systems. Whereas the Anglo-Saxon scientific literature often speaks of ‘disorder’, Dutch and German literature tends to use words such as ‘disturbance’ or ‘dysregulation’ when referring to dysfunction or disease. Such use of words speaks to a general intuition that disease and other forms of maladaptive behavior somehow involve a problem of control and a loss of ‘order’. In the previous section, we showed that the emergence of disorder may be a generic feature of open dissipative systems that overload with free energy and reflect a loss of central integrative governance [27,36]. The ubiquitous presence of rising disorder levels, tipping points, and other critical phenomena in living systems under difficult conditions suggests that many forms of malfunction and disease involve a generic mechanism (see Section 1). We therefore propose that any physical, mental, or social disorder eventually involves a loss of integrative control due to an excess of free energy (stress, prediction error). The ensuing overflattening of goal hierarchies then causes suboptimal inference and maladaptive behavior (see above). The cascading failure of hub structures is a key element in our theory and is increasingly being recognized as an important factor in the emergence of physical and mental disorders. Examples involve a cascading failure of hub genes in metabolic disease [136] and cancers [137], hub cells in diabetes mellitus [116], and hub brain regions in neurological disease [115] and mental disorders [138]. Studies have shown that similar processes govern the collapse of social hierarchies and the emergence of social disorder in animal and human societies (see below). Nevertheless, this theory remains to be tested by systematically examining (permutation) entropy scores and other hallmarks of critical slowing down as a function of the hierarchical depth of goal hierarchies in a diverse range of living systems under severe levels of stress. Due to the ethical difficulties of such studies, a valuable approach is to test these assumptions in silico, by systematically examining changes in signal transduction and overt behavior of hierarchical Bayesian control systems, e.g., using hierarchical machine learning techniques. In our recent paper, we made several recommendations for such studies [21].

Although disordered states tend to be undesirable in organisms, this does not mean that order is always good and disorder is always bad. As stated above, signal transduction in organisms is normally poised on the edge between order and disorder, reflecting optimal information processing. Some level of chaos (disorder) is, therefore, required for organisms to respond in a lively and creative fashion to environmental challenges [133]. Overly ordered states may on the other hand produce malfunction, e.g., when overly controlling hierarchies exert too much influence over hierarchical message passing at lower hierarchical systems and cause inflexible states of low adaptability. Eventually, however, any problem in the balance between order and disorder is likely to produce high levels of prediction error that cause organisms to ‘lose control’ and system dynamics to tilt heavily toward ‘disorder’.

Since prediction error (stress) can be defined as the difference between a prediction and an actual perception, it is fundamentally a relative measure. This means that the cause of stress may lie either with the individual, since it expresses some rare or extreme setpoints (encoding rare or extreme niches that are difficult to occupy), or with the environment, which may itself be so rare or extreme that that it does not fit otherwise frequently expressed setpoints. In both cases, stress may increase to such levels as to cause goal hierarchies to collapse and disorder to emerge. For example, thermophilic or acidophilic bacteria may thrive in hot-water springs or extremely acid conditions, but fail to thrive under more common conditions that would otherwise be considered favorable for most organisms. Conversely, most organisms that encode quite common environmental niches as world models will express high levels of prediction error in response to evolutionary ‘unfamiliar’ stressors such as toxins or ultrahigh temperatures. This shows that the concepts of stress and disorder that we propose are fundamentally relative: one set of priors (thermostats, goals, world models) may cause an individual to have a nice fit with its current environment and remain stable, whereas the same set of priors may produce stress and disorder in some other niche. The relativity of stress and disorder, however, does not detract from the objectivity with which their presence can be established.

Since a loss of integrative control may explain the emergence of disorder across scale levels, we will now examine how it applies to the specifically human perspective, by discussing how stress may produce disorder at intraindividual, interpersonal, and population levels. These scale levels are the main focus of psychiatry as a medical discipline, with its traditional focus on biological, psychological, and social determinants of mental illness [139]. This represents a novel approach, and the examples that are given can be read as avenues for further research.

## 10. The Human Perspective: Disorder at the Individual Level

Just like woodlice, humans can be modeled as hierarchical Bayesian control systems with goal hierarchies that encode the econiches they wish to explore. The major difference is that human world models are more sophisticated, which allows them to encode complex econiches at high levels of parsimony and abstraction (see above). Since humans are a highly social species, their goal hierarchies often encode social goals (e.g., partners, jobs, and social positions), and stress often involves social stress (e.g., not finding a suitable partner or job, or not reaching some social position in time). Where people fail in the pursuit of such goals, stress and disorder may emerge.

Within the field of psychiatry, is has been known for some time that there are at least two distinct types of mental disorders. One involves episodic disorders, which represent a temporary decline in mental abilities with respect to a previously attained level of functioning (e.g., panic attacks, major depression, or psychotic episodes). Such disorders typically emerge and resolve at relatively discrete moments (e.g., within hours or days), indicating the presence of tipping points [4,5]. Another type of psychiatric problems involves trait disorders, in which patients exhibit a series of stable mental traits that together increase the risk of episodic disorders across longer timeframes (e.g., avoidant, dependent, or borderline personality profiles). With respect to acute or episodic disorders, is has been proposed that such disorders represent various forms of ‘false inference’, i.e., a suboptimal balance between top-down predictions and bottom-up belief updating by prediction errors [140]. Interestingly, this overall balance between predictions and prediction errors is controlled by the ‘precision’ of such signals, i.e., their signal-to-noise ratio, which expresses the overall level of ‘confidence’ that the information conveyed by the signal is correct (see above). On the one hand, such problems of inference may involve the emergence of ‘hyperprecise priors’, which are models that are overly dominant in suppressing prediction errors and leave little room for alternative explanations of the observed events (this could explain the occurrence of e.g., hallucinations, delusions, phobias, and other anxiety disorders). On the other hand, prediction errors may become overly precise, signaling high confidence that some signal carries consistent uncertainty and leaving little room for systems to converge upon a suitable explanation of observed events (this may explain, e.g., feelings of dissatisfaction, emptiness, pathological doubt, and obsessive–compulsive behavior) [140]. Note that the same mental problems can be explained by presuming *hypo*precise priors and hyperprecise prediction errors: all such variants are likely to exist in the form of (epi)genetic variations in neurotransmission and cytoarchitecture, which may explain different subtypes of mental disorders [141]. As observed, high levels of stress cause a net decrease in precision levels in living systems, which may modulate the precision balance and cause suboptimal inference. In the human brain, the precision of signals is controlled by neuromodulatory neurotransmitters such as serotonin, noradrenaline, dopamine, and acetylcholine [140]. Most neurotropic drugs that are used in psychiatry modulate the release of such neurotransmitters, which may be beneficial in correcting the precision balance and reducing symptoms [142].

A problem of the precision balance provides a likely explanation for various forms of psychopathology, but does not in itself explain the episodic versus chronic nature of such phenomena [21]. We, therefore, propose that episodic disorders result from a (temporary) collapse of goal hierarchies in response to stress, whereas trait disorders result from a failure of such hierarchies to develop normally. In episodic disorders, a cascading failure of a goal hierarchy may reduce integrative control until the system passes the edge of chaos, producing tipping points and disorder. This can be compared to a cascading failure of a multilevel thermostat which then gives off the wrong values, causing problems with heating the house (producing maladaptive behavior). In trait disorders, on the other hand, people may inherit or acquire a set of priors (thermostat settings) that encode a predilection for certain (social) econiches. When such prior settings do not match the actual state of the environment, prediction error (stress) and disorder may emerge. For example, people may differ with respect to their desire to explore new surroundings or to avoid negative outcomes. When the environment matches such predilections (e.g., the adrenaline seeker at the edge of the Grand Canyon, or the couch potato in front of the TV), prediction error and ‘stress’ are minimal, and disorder is some distance away. When the opposite is true (i.e., the adrenaline seeker sitting on a couch and the couch potato living on the edge), fitness is poor, and disorder may emerge. People with extreme prior settings (‘temperaments’) can in this respect be compared to extremophile bacteria that thrive in extreme environments but not in others or to central heating systems with high thermostat values. Such systems perform well in hot climates but overheat and break down in colder climates, since they are unable to reach some extremely high goal temperature. The more rare or extreme such prior settings are, the more difficult it will be for an individual to find econiches that are equally rare or extreme. Niche exploration may, therefore, take a long time and, consequently, chronic prediction error will occur (i.e., chronic stress). This increases the chances of collapsing goal hierarchies and episodic disorders.

Fortunately, people do not simply inherit a fixed set of priors which they have to deal with throughout the rest of their lives. The innate set of priors is tuned by a continuous process of belief updating, which allows them to meet environmental conditions halfway. Moreover, people may gain additional (allostatic) levels of control over their innate priors through the vertical outgrowth of their goal hierarchies. This involves the addition of hierarchical layers to a hierarchical control system over the course of individual development [143]. Belief updating within these successive hierarchical layers globally corresponds to Pavlovian, habit- and goal-directed learning [20]. Thus, people ‘grow’ a set of world models that encode increasingly sophisticated (social) econiches, which globally involve internal (self), external (social), and crosscutting (normative) models. Together, such high-level models may be referred to as ‘character’, and the combination of temperament and character maturation is called ‘personality development’ [144,145]. Character development may allow people to find a suitable (social) niche after all, even when their innate set of priors (temperament) is rare or otherwise extreme. When character development fails for any particular reason, this results in less sophisticated world models that will cause people to seek out suboptimal (social) econiches (i.e., show maladaptive behavior). Such shallow world models are more likely to collapse during stress and reach a hierarchical depth below which the system tips toward an undercontrolled state of disorder. This would be a testable model of the emergence of episodic disorders or ‘crises’ in patients with traits disorders such as ADHD, autism spectrum, or personality disorders.

The specific phenomenology that ensues in various mental disorders can be further explained by observing the general architecture of hierarchical Bayesian control systems (Figure 5 and Figure 6). Depending on the location and depth of the collapse of such structures under stress, different symptoms may be produced [21,140,146]. Since stress preferentially affects the integrative top of a goal hierarchy, a top-down collapse from goal-directed to habitual, instinctual, or even reflexive behavior may generally be observed in episodic disorders (Section 6). This may explain why a decline in self-functioning, interpersonal functioning, and/or normative functioning (a collapse of high-level goal-directed functions) is a common hallmark in different forms of mental illness, whether involving episodic or trait disorders (Figure 7, Section 7) [147,148]. Since the functional integration of specialized brain regions is important for maintaining a sense of awareness and proper cognition [149,150], the functional segregation produced by collapsing hierarchies may explain a loss of awareness with respect to self-referential, social, or transpersonal goals. When internal (self)models become less sophisticated or precise, people report difficulties experiencing a coherent sense of self. Depending on the depth of such a deficit, this may involve symptoms that vary from a lack of agency or autonomy to a sense of depersonalization, disintegration, or dissociative disorder [151,152,153]. When external (social) models are involved, people may become unaware of the needs and intentions of others (have difficulty mentalizing). This may cause frequent misunderstandings, inspire paranoid interpretations of events, or prevent individuals from experiencing a sense of communion (i.e., showing interest in others, caring for and trusting other people). When crosscutting (normative) models are involved, people may show a reduced ability to feel connected across larger individual differences and timeframes (generations) or have the experience that life lacks inherent meaning: a state that is called ‘demoralization’. Demoralization appears to be present in nearly all forms of mental illness and is arguably the most important reason for people to seek treatment [154]. This could be explained by the fact that stress causes the highest regions of a goal hierarchy to collapse first, which we propose harbors a crosscutting (normative) hierarchy that is responsible for generating our ‘highest goals’. A collapse of such high-level structures may then produce problems further down the hierarchy. For instance, a failure or disinhibition of input (perceptive) hierarchies may produce hallucinations and other perceptual distortions, and a disinhibition of affective hierarchies may produce anxiety or mood disorders, whereas, when output (action control) hierarchies are involved, this may produce problems with executive functions (e.g., a loss of praxis and disorders of motor or endocrine planning).

To summarize, a hierarchical taxonomy of psychiatric disorders can be drafted that can be linked to suboptimal inference at different scale levels and locations within a hierarchical Bayesian control system. This idea relates strongly to one of the leading alternatives to the traditional (categorical) taxonomy of psychopathology as formulated in the Diagnostic and Statistical Manual for Mental Disorders (DSM-5): the hierarchical taxonomy of psychopathology (HiToP) [155]. The firm rooting of active inference in neuroscience and biology holds promise for integrating another alternative classification system (RDoc) into clinical practice, which puts more emphasis on the neurobiological underpinnings of psychiatric disorders [156]. According to DSM-5, a set of mental states and traits qualifies as a disorder if a certain set of mental states interferes ‘in a significant way’ with everyday personal functioning (e.g., maintaining relationships, managing a job, or performing activities of daily living). This introduces a degree of subjectivity to the definition of ‘disorder’ that is quite valuable, since objective measures may ignore aspects of subjective experience that may be crucial for determining the level of personal suffering. On the other hand, such subjectivity makes it difficult to quantify and compare mental states. We, therefore, propose to use permutation entropy as an objective disorder criterion, which can be used to link ‘disorder’ at different levels of biological organization to subjective experience and personal suffering. This may include the calculation of permutation entropy scores at level genetic, neurophysiological, psychometric, social, or demographic scales in order to quantify disorder at various levels (see Section 1).

At this point, it is important to note that disorder cannot always be measured within the individual itself, but rather within the environment that surrounds the individual: so-called ‘internalizing individuals’ have a tendency to model the hidden cause of experienced prediction errors within themselves and to engage in self-corrective activity in order to solve such errors (e.g., through a revision of their assumptions or by acting in response to the presumed internal deficit) [157]. In such a case, any stress or disorder is more likely to accumulate within the individual itself and take the form of a psychiatric disorder. In contrast, externalizing people tend to project the hidden cause of experienced errors outside of themselves and to reduce prediction errors by performing actions that are aimed at correcting the presumed external problem (with relatively little belief updating of their self-models). In that case, stress and disorder are more likely to accumulate in the environment rather than in the individual itself [157]. Our model, therefore, shows that people may still ‘have a problem’ even if they themselves do not show any signs of stress or disorder, since they induce a lot of disorder in their environments. This departs from the current disorder criterion as formulated in the DSM-5, which states that, in order to qualify as a disorder, a mental phenotype must occur “within an individual” and cause “clinically significant distress or disability” [158]. A more relative definition of mental disorder would, therefore, include ‘stable people’ that always sleep well but meanwhile produce unsophisticated models and corresponding actions that leave their environments in a state of complete uproar. This example illustrates the fact that the maladaptive behavior of one individual may pass on to other individuals, corresponding to a scale transition. This is discussed in the next section.

## 11. The Human Perspective: Disorder at the Interpersonal Level

**Figure 9:**
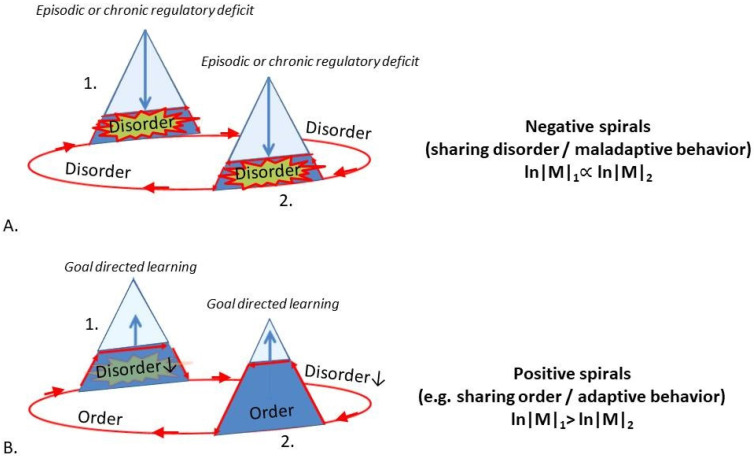
The spread of disorder through social networks as a function of hierarchical control. *Disorder may spread through social networks as a function of the amount of hierarchical control. Two special cases of mutually reinforcing social interactions are shown. (**Panel A**) The social clonus. This is a self-propagating (circularly causal) action–perception cycle between people (or communities of people) that is caused by a loss (or lack) of central integrative processing, e.g., during episodic disorders or in personality disorders. The unpredictable responses (maladaptive behavior) produced by individual/population 1 serve as input to individual/population 2 that similarly lacks the ability to view such behavior in a broader context. This results in a maladaptive response that feeds back to individual 1, which raises stress and disorder levels within individual 1, and the cycle repeats. Social clonuses may generalize to larger social communities via collateral connections, producing disorder at a population level. Ln|M| = whole system permutation entropy. (**Panel B**) Improving hierarchical control (e.g., by recovering from an episodic disorder or promoting the outgrowth of sophisticated goal hierarchies (personality development)) puts an intrinsic break on the spread of maladaptive behavior (disorder) though social networks. See text for details*.

Recent studies have shown that the free-energy principle can be used to model information transfer in social networks of animals and humans (i.e., communication patterns) [159,160]. A model has been proposed in which one individual monitors the behavioral output of another in order to infer the hidden common causes behind the observed behavior (i.e., its meaning or intentions). In order to read their mutual intentions, organisms must synchronize their responses, which in this view defines a social tie. Predicting the intentions behind another person’s behavior becomes increasingly difficult when the observed behavior becomes increasingly unpredictable. This may happen when a subject’s world model flattens to the point where the corresponding behavior of the individual loses its hierarchical correspondence with the actual state of the world. Such ‘maladaptive behavior’ is marked by high levels of permutation entropy (disinformation, low levels of predictability, see Section 1). This can be the case, e.g., in psychiatric patients with affective or psychotic disorders, in which the connection between the outside world and observed behavior does not seem to make sense (i.e., is unpredictable). When the behavioral output of some individual is sufficiently unpredictable (maladaptive), this may raise prediction error (stress) levels in another individual to the point where it causes the goal hierarchy of this new individual to collapse and the individual to show unpredictable (maladaptive) behavior of his own. Such ‘disorder’ may consequently be conveyed upon yet other individuals or feed back to the first individual to form a closed loop. Thus, disorder (disinformation) may spread through social networks (Figure 9). In an extreme example, individual 1 may be highly annoyed by the loud music produced by individual 2 (their stressed-out neighbor). This raises stress levels to the point where it causes a collapse of hierarchical control in individual 1, who is subsequently unable to factor in the needs of individual 2 (e.g., pay them a visit when they need help). Based on their decontextualized models, individual 1 then decides to make some noise of their own, keeping individual 2 (and perhaps some others) awake and removing any residual levels of control that individual 2 might have. Individual 2 then gets back at individual 1, etc. Thus, people may hold each other captive in complex webs of underregulated reflex arcs that are self-sustaining and difficult to extinguish, since they are insufficiently suppressed by more sophisticated (contextualized and socially inclusive) world models (Figure 9A). This can be compared to a neurological clonus, which is a disinhibited reflex that sustains itself by means of its own motor response, which serves as a trigger for a novel response. Such pathological reflexes are caused by the disappearance of higher-order regulatory functions (e.g., by a tumor or an infarction) that normally suppress the primary reflex arc. Similarly, the ‘social clonus’ may cause strong loops in social relationships (such as intense interpersonal conflict or symbiotic relationships) due to a lack of top-down regulatory constraint. Indeed, several studies have shown that emotional states such as (un)happiness and loneliness or mental illness such as major depression may spread through social networks in ways that are analogous to infectious disease, although a general mechanism for such ‘social contagion’ seems to be lacking [161]. The free-energy principle may explain such effects in terms of the spread of (dis)information through social networks in the context of insufficient hierarchical control.

The above is an extreme example of how a collapse of goal hierarchies may cause disorder or disinformation to spread through social networks (either in tight social loops or in wider social communities). A more delicate transmission of disinformation may take place in less extreme situations, e.g., when goal hierarchies are only mildly underdeveloped, as in personality disorders or intellectual deficits. Such ‘shallow’ world models may produce subtle forms of maladaptive behavior, which may only slightly raise disorder (disinformation) levels in other individuals, causing social networks to become slightly noisier. In short, the transmission of disorder (unpredictability) through social networks, as well as the emergence of vicious cycles between people, is a function of the hierarchical depth of all goal hierarchies that lie along the traveled path. A natural resistance to such spread would, therefore, be to encourage individuals to develop mature and contextually rich goal hierarchies (i.e., by recovering from acute mental illness, or through education or psychotherapy; Figure 9B). The fact that people form social ties that are based on the predictability of their responses highlights the importance of a shared normative set in the form of an overarching predictive model, which promotes social connectivity across large individual differences by emphasizing communalities between people [106,107,159]. Without such high-level constraint, self-propagating patterns of disorder may eventually generalize to population levels, where large groups of individuals enter into a collective state of disorder (e.g., lingering conflicts or war). This is discussed in the next section.

## 12. The Human Perspective: Disorder at a Population Level

By now, many studies have shown that the ‘scale-free’ principles of network architecture and function that govern living systems at different scale levels of organization also apply to social networks. Scientists have long been fascinated by the *small world* structure of social networks that allow any two persons on this earth to be connected through an average of only six degrees of separation [162]. Just like living systems at smaller spatial scales, social communities are held together by a limited number of hub individuals such as kings and queens, presidents, CEOs, pop idols, influencers, news readers, professors, schoolteachers, and social workers. Large social networks consistently show a nested modular (hierarchical) information bottleneck structure, just like network systems at a molecular and cellular levels [163,164]. This suggests that some parts of social networks are dedicated to input (perception), throughput (goal setting), and output (action) of whatever messages are passed between individuals. Social networks also display dynamic phenomena that resemble features of hierarchical message passing in living systems, such as oscillations, bursts, and tipping points that define the spread of infections, mass psychosis, mass hysteria, or riots [161,165,166,167]. Such processes are increasingly studied from a biophysical perspective, sparking the existence of a new field called computational sociology [168,169,170]. The many parallels that exist between signal transduction within single organisms and information transfer within social networks have led scholars to reserve the term ‘superorganism’ for some of these collectives (such as ant and termite colonies, beehives, and communities of blind mole rats). Although humans generally show a larger level of individual autonomy than the individual agents of a superorganism, it has been argued that human collectives can flexibly behave as superorganisms under certain conditions (e.g., [171]).

Despite such findings, however, the question remains whether the analogy with multicellular organisms ends here, or whether social systems are indeed involved in some form of active hierarchical Bayesian inference. In order to answer such questions, future studies may want to examine whether a division of labor exists between individuals that act primarily as priors (e.g., issuing hypotheses) and those that act as prediction error units (issuing deviations from these hypotheses). For instance, scientists or defense lawyers may be engaged in circularly causal dynamics of hypothesis generation and falsification. When compared to other living systems, however, human individuals are more likely able to flexibly shift their social roles as priors or prediction error units depending on the topic discussed. At a larger spatial scale level, the bowtie structure of social networks suggests a global division of labor across collective perception, goal setting, and action control. It may, therefore, be worthwhile to study the distribution of social roles and professions across these global domains of the social network. For instance, global perception may be shaped by journalists, scientists, and other influencers that feed the collective with novel information and facts (input). Collective goal setting may involve a legislative power that processes such information at a more abstract level to draft new laws (i.e., a hierarchy of priors). These are then criticized and updated by a house of representatives (i.e., a hierarchy of prediction errors), after which a judiciary power applies these updated laws to issue out policies (action control). The executive branch (output) then enforces these laws onto the environment (e.g., soldiers and police). In this model, the departments of internal and external affairs are involved in generating self-models and social models at the level of nation states, whereas crosscutting (or normative) models may be formed by some philosophical or religious institute of power.

Studies show that social networks may display cascading failures of social hierarchies in response to high levels of interpersonal traffic (e.g., a collapse of the social chain of command) [120,121,165]. In stressful situations, a mild flatting of a social hierarchy may be an adaptive response of social systems in times of crisis. This may speed up response times of collective decision making by bypassing elaborate processing at the top of social hierarchies (e.g., throughout history, a ‘strong man’ was appointed in times of crisis to force certain decisions through parliament). However, an overflattening of a social hierarchy may produce a state of disinhibited disorder within its lower ranks [120,121,165]. At a higher-scale level of social organization, a collapse of integrative government may cause the functional segregation of social communities and individuals, leading to increased polarization and interpersonal conflict [172]. This corresponds to a state of suboptimal inference of collective goal states and the production of maladaptive behavior at group level. As anywhere else in living nature, such changes should translate into rising levels of permutation entropy in hierarchical message passing (e.g., Twitter messages or other social media). We, therefore, argue that ‘losing control’ is basically the same process anywhere, whether involving bacteria succumbing to antibiotics, people developing physical or mental disorders, or social systems slipping into civil war. Permutation entropy may be a universal way to quantify disorder in timeseries at each of these scale levels of biological organization and to take the necessary precautions.

## 13. Conclusions

We reviewed the concept of permutation entropy as a universal disorder criterion. The allostatic overload and cascading failure of living systems and the emergence of disorder in response to stress appears to be a special case of the functional or structural disintegration of open dissipative systems as a universal response to a free energy overload. When confirmed in experimental studies, physical, mental, and social disorders can be described, predicted, and understood using the same mathematical language. This unifying principle may help to promote collaboration amongst a diverse range of disciplines and urge scientists to push forward a common research agenda that may speed up discoveries in all relevant fields.
